# Studies on the interaction of isocyanides with imines: reaction scope and mechanistic variations

**DOI:** 10.3762/bjoc.10.3

**Published:** 2014-01-06

**Authors:** Ouldouz Ghashghaei, Consiglia Annamaria Manna, Esther Vicente-García, Marc Revés, Rodolfo Lavilla

**Affiliations:** 1 Barcelona Science Park, University of Barcelona, Baldiri Reixac 10–12, 08028 Barcelona, Spain; 2 Laboratory of Organic Chemistry, Faculty of Pharmacy, University of Barcelona, Av. Joan XXIII sn, 08028 Barcelona, Spain

**Keywords:** azetidines, heterocycles, imines, isocyanides, multicomponent reactions

## Abstract

The interaction of imines with isocyanides has been studied. The main product results from a sequential process involving the attack of two units of isocyanide, under Lewis acid catalysis, upon the carbon–nitrogen double bond of the imine to form the 4-membered ring system. The scope of the reaction regarding the imine and isocyanide ranges has been determined, and also some mechanistic variations and structural features have been described.

## Introduction

**Scheme 1:**
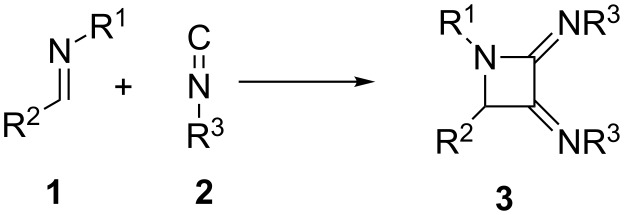
Azetidine formation from the interaction of imines with isocyanides.

The interaction of imines with isocyanides is mainly focused on to the well-known Ugi multicomponent reaction (MCR) [1]. This fundamental process features the participation of a carboxylic acid group which attacks the intermediate nitrilium ion thus leading, after the Mumm rearrangement, to α-amidoamides. However, the direct reaction of imines and isocyanides has been considerably less studied and, in the absence of a carboxylate, the mechanistic outcome is considerably modified [2]. A relevant precedent was described by Deyrup in the late sixties, demonstrating the double incorporation of an isocyanide moiety to an imine [3–4]. Interestingly, the 3CR between a carbonyl, an amine and an isocyanide, taking place through the intermediacy of the in situ generated imine, leads to α-aminoamidines, resulting from the trapping of the nitrilium cation by the remaining amine [5–8]. Taking into account the intrinsic interest in the azetidine scaffold in medicinal chemistry [9], we decided to study in detail the formation of bis(imino)azetidines **3** from the interaction of imines **1** and isocyanides **2** (Scheme 1), including the scope of the reaction and mechanistic features of this interesting ABB’ process [10].

## Results and Discussion

### Reaction scope

In this section we analyze the reaction conditions, the structural features of the products and the scope of the reactants.

### Reaction conditions

**Scheme 2:**
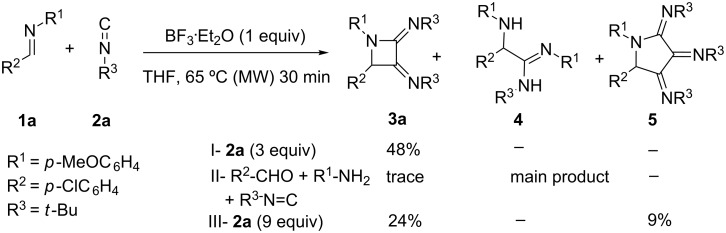
Reaction conditions.

We began our studies with the experimental screening of the solvents, catalysts and temperatures suitable for this transformation. In this respect, taking imine **1a** (R^1^ = *p-*MeOC_6_H_4_; R^2^ = *p-*ClC_6_H_4_) and isocyanide **2a** (R^3^ = *t*-Bu), we tested the standard reaction in THF, MeCN, and CH_2_Cl_2_ as solvents using a variety of Lewis and Brønsted acid activating agents (20–100 mol %) including: InCl_3_, Sc(OTf)_3_, AuCl_3_, AgOTf, GaCl_3_, NbCl_5_, camphorsulfonic acid, I_2_, Br_2_·SMe_2_ and BF_3_·OEt_2_, at temperatures ranging from rt to 80 °C. The transformations were tested under standard heating or microwave irradiation, with reaction times lasting form 30 min to 48 h. The imine **1a** was generated in situ, using MS 4 Å, or previously prepared by condensation of the corresponding aldehyde and aniline. It was found that the best conditions were obtained using BF_3_·Et_2_O as the activating agent in stoichiometric amounts in THF, at rt for 24 hours or under MW irradiation for 30 min at 65 °C, allowing the formation of the expected azetidine **3a** in 43% and 48%, respectively. Compounds **4** and **5** could not be detected (Scheme 2). When the process was run as a true MCR (mixing the amine, the aldehyde and two equivalents of isocyanide **2a**), the adduct **3a** was produced in trace amounts and the main product was the α-amino-amidine **4**, in good agreement with previous reports [5–8]. In a different experiment, the addition of a 9-fold excess of isocyanide **2a** to the imine **1a** under the usual conditions led to detection of tris(imino)pyrrolidine **5** (9%) as the minor product, and azetidine **3a** as the major component (24%, Scheme 2).

### Structural elucidation

**Figure 1:**
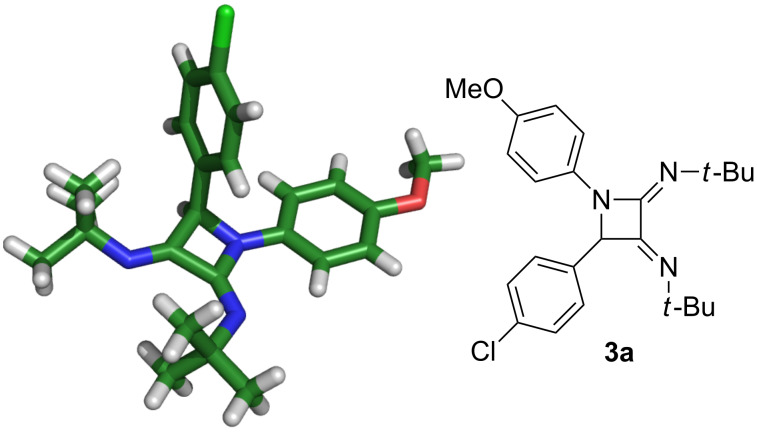
X-ray diffraction analysis of azetidine **3a**.

Although we could confirm the constitution of the azetidine **3a** by spectroscopic methods (NMR, MS), the stereochemistry of the C=N bonds present in the structure remained unsolved. Furthermore, no conclusive nOe’s were observed to assign these stereogenic centers, and there were no reports in the literature regarding this point. A monocrystal of the bis(imino)azetidine **3a** was subjected to X-ray diffraction analysis and the solid state structure is depicted in Figure 1 [11].

**Scheme 3:**
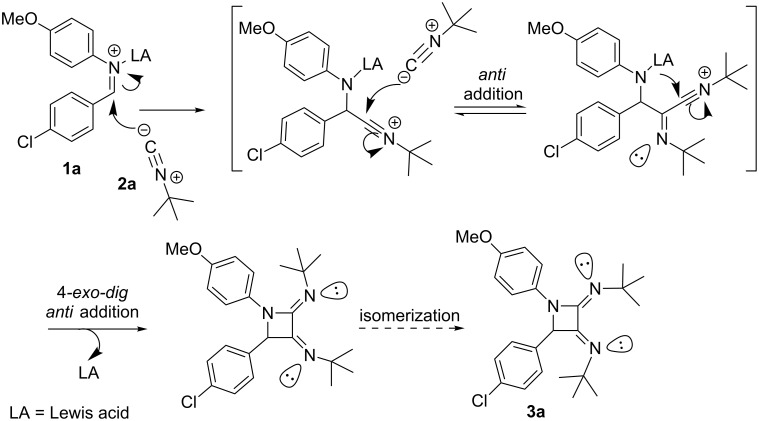
Stepwise mechanism for the formation of azetidine **3a**.

This result confirms the structural features associated to this scaffold, and also raises some questions on the origin of the stereochemistry associated to the C=N moieties. First of all, the process can be explained by an attack of one isocyanide equivalent to the Lewis acid (LA)-activated imine, leading to a first nitrilium intermediate (Scheme 3), then a subsequent attack would give raise to a second nitrilium ion [12–15], which is trapped by the nucleophilic nitrogen of the imine. This last step is formally a disfavoured nucleophilic 4-*exo-dig* process [16], although recent results and calculations show that it should be feasible and some examples have been disclosed [17]. Interestingly, the expected *anti*-addition mode towards the carbon–nitrogen triple bonds should generate *Z* configurations [18–19], which are not observed in the solid-sate, thus suggesting isomerization processes affecting the C=N moieties, likely mediated by acid-catalyzed prototropy or other tautomerization steps. Computational calculations (MMFF, AM1 and BL3YP/6-31G* performed in a Spartan suite) suggest that the differences in the heat of formation among some geometrical isomers are small. Furthermore, NMR spectra nearly always display a single set of signals, thus discarding further isomerization events once the compounds are detected or isolated.

### Reactant scope

Next, the scope of the reaction was investigated, and a variety of imines was subjected to the interaction with a range of isocyanides under the optimized conditions to determine the generality of the process and to detect possible restrictions. Results are depicted in Table 1.

**Table 1:** Scope of the imine and isocyanide starting materials.

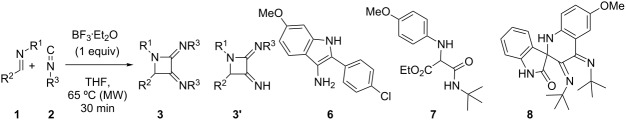				

entry	R^1^	R^2^	R^3^	yield

1	4-MeOC_6_H_4_	4-ClC_6_H_4_	*t*-Bu	**3a** (48%)
2	4-MeOC_6_H_4_	4-ClC_6_H_4_	*c-*C_6_H_11_	**3b** (41%)^a^
3	4-MeOC_6_H_4_	4-ClC_6_H_4_	Bn	**3c** (63%)^a^
4	4-MeC_6_H_4_	4-ClC_6_H_4_	4-MeOC_6_H_4_	**3d** (19%)^b^
5	3-MeOC_6_H_4_	4-ClC_6_H_4_	*t*-Bu	**3e** (19%) + **6** (27%)
6	4-MeOC_6_H_4_	2-ClC_6_H_4_	*t*-Bu	**3f** (12%) + **3f'** (15%)
7	2-MeC_6_H_4_	4-ClC_6_H_4_	*t*-Bu	**3g** (31%)
8	*n-*C_3_H_7_	4-ClC_6_H_4_	*t*-Bu	**3h'** (32%)
9	4-MeOC_6_H_4_	EtOCO	*t*-Bu	**3i** (9%)^a^ + **7** (34%)
10	4-MeOC_6_H_4_	EtOCO	*c-*C_6_H_11_	**3j** (34%)^a^
11	4-MeOC_6_H_4_	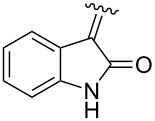	*t*-Bu	**3k** (14%)^b^ + **8** (17%)
12	4-MeC_6_H_4_	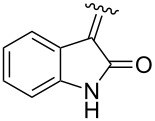	*t*-Bu	**3l** (28%)
13	4-MeOC_6_H_4_	4-ClC_6_H_4_	Ts-CH_2_	–
14	4-MeOC_6_H_4_	4-ClC_6_H_4_	MeO_2_C-CH_2_	–

^a^The reaction was performed at rt. 20% of catalyst was used. ^b^The adduct could not be isolated in pure form.

The scan of isocyanides shows that their nucleophilicity plays a determining role in the reactivity, since *tert*-butyl, cyclohexyl and benzyl isocyanides (Table 1, entries 1–3, 5–12) show a relatively high conversion, whereas the aromatic isocyanide was less reactive (entry 4) and the weaker nucleophiles TOSMIC and methyl isocyanoacetate were not productive (Table 1, entries 13 and 14) [20]. Additionally, with respect to imines, the reaction works well for substrates generated from aromatic aldehydes and anilines displaying *o*-, *m*- and *p*-substituents (Table 1, entries 1–7). Moreover, in one case the 3-aminoindole **6** was detected (27%), in agreement with a recent report (Table 1, entry 5) [21].

*N*-Alkylimines seem to react appropriately (Table 1, entry 8). Furthermore, we have observed some *tert*-butyl eliminations, probably due to competing reactions under the acidic conditions (Table 1, entries 5, 6 and 8). In general, imines containing electron-rich *N*-aryl moieties showed higher reactivities, and we were not able to isolate azetidine adducts **3** from the reaction of arylimines containing strong electron-withdrawing groups at the aniline moiety (*p*-CF_3_, *p*-F and *p*-COOEt), likely because of their low conversions. However, the presence of such groups linked to the carbon of the imine did not seem to disturb their reactivity (Table 1, entries 1–8). Interestingly, the reaction of glyoxylate imine (Table 1, entry 9) with *tert*-butyl isocyanide led to the formation of minor amounts of the azetidine adduct **3i** (9%) whereas the α-aminoamide **7** (34%) was the major component. The formation of amidoamides has been reported in the *p*-toluenesulfonic acid-catalyzed interaction of anilines, amines and isocyanides [8]. On the other hand, the reaction of the same imine with cyclohexyl isocyanide gave azetidine **3j** (34%) in a selective manner (Table 1, entry 10), without traces of the corresponding α-aminoamide. Different types of activated substrates displaying C=N bonds (*N*-sulfinylimines, oximes and hydrazones) were studied, but none of them reacted productively with isocyanides under the described conditions. Finally, the reaction with isatinimines led to the formation of the spiro-azetidines **3k** (14%) and **3l** (28%, Table 1, entries 11 and 12). Remarkably, in the former case an intramolecular cyclization of the nitrilium ion upon the electron rich *p*-methoxyphenyl group took place and led to the formation of the bis(imino)tetrahydroquinoline **8** (17%, Table 1, entry 11).

### Mechanistic analysis

**Scheme 4:**
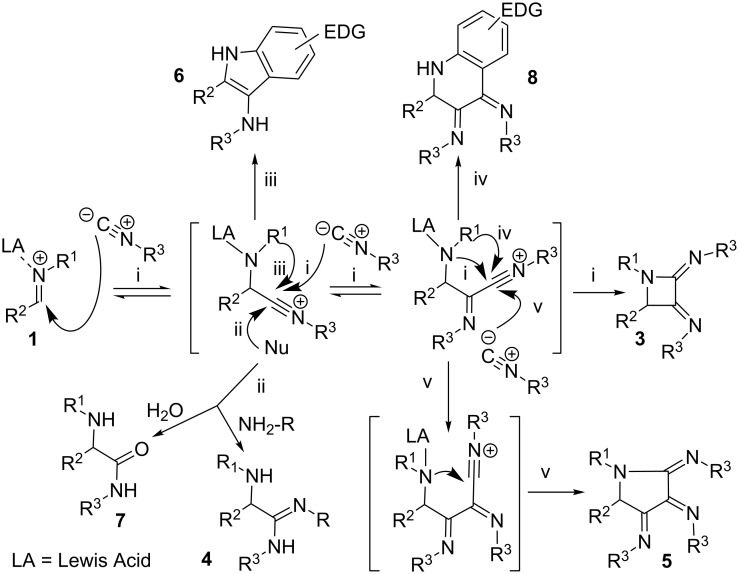
Manifold reaction mechanism.

Taking into account the structural variety observed in this family of reactions, a rational explanation is needed to understand the formation of such products. Here we describe a simplified hypothesis based on the well-known nucleophilic addition of isocyanides to Lewis acid-activated imines (Scheme 3). The first nitrilium intermediate can evolve through a second addition and ring-closure to yield the azetidine adduct **3** (Scheme 4, route i), or can also be trapped by water or amine/imine nucleophiles leading to α-aminoamides **7** and α-aminoamidines **4**, respectively (Scheme 4, route ii). The formation of aminoamide **7** was restricted to the use of glyoxylate imines, and happens only with *tert*-butyl isocyanide, but not with cyclohexyl isocyanide. On the other hand, the aminoamidines **4**, the standard adducts from the amine–aldehyde–isocyanide 3CR were observed in some occasions [5–7] under our conditions presumably by attack of the unreacted imine upon the nitrilium cation. These facts suggest that either a good nucleophile (amines, imines) may act intermolecularly to undergo fast addition to this intermediate or, when an alkyl carboxylate group is present it may stabilize the nitrilium intermediate precluding further addition events and leading to the aminoamides **7** after the final aqueous treatment.

Furthermore, we have detected indole **6** arising from the cyclization of electron-rich aromatic rings linked to the imine nitrogen upon the electrophilic nitrilium intermediate, in agreement with a Sorensen report (Scheme 4, route iii) [21]. Finally, the imino-nitrilium cation can be trapped by an aromatic ring when using isatin imines, leading to bis(imino)tetrahydroquinoline **8**, (Scheme 4, route iv). In a reaction using a large isocyanide excess, a triple insertion of the isocyanide moiety has been observed, the adduct being the tris(imino)pyrrolidine **5** (Scheme 4, route v).

## Conclusion

As a summary, we have described structural and mechanistic features for the bis(imino)azetidines arising from the imine–isocyanide interaction, finding that the process involves a sequential double isocyanide incorporation into the C=N bond. The final step is a nucleophilic 4-*exo-dig* cyclization, and the anti addition modes likely lead to less stable stereoisomers which spontaneously isomerize to the observed compounds. Furthermore, we have determined the scope of the reaction, according to the imine and isocyanide starting materials, and a small collection of multicomponent adducts has been prepared. These structures bear a novel azetidine scaffold of potential interest in medicinal chemistry [22–23]. Although the yields are modest, the compounds can be conveniently prepared in a straightforward manner. A part of the azetidine structure distinct scaffolds have been obtained from the interaction of different reactant combinations: α-aminoamides, α-aminoamidines, indoles, bis(imino)tetrahydroquinolines and tris(imino)pyrrolidines. Finally, a unified reaction mechanism that can account for the production of this rich structural outcome has been proposed.

## Supporting Information

File 1 Experimental procedures, characterization data, copies of the NMR spectra for all new compounds and X-ray views of azetidine **3a**.

## References

[1] Dömling A (2006). Chem Rev.

[2] El Kaïm L, Grimaud L, Nenajdenko V G (2005). Isocyanide Chemistry.

[3] Deyrup J A, Vestling M M, Hagan W V, Yun H Y (1969). Tetrahedron.

[4] Morel G, Marchand E, Malvaut Y (2000). Heteroat Chem.

[5] McFarland J W (1963). J Org Chem.

[6] Keung W, Bakir F, Patron A P, Rogers D, Priest C D, Darmohusodo V (2004). Tetrahedron Lett.

[7] Khan A T, Basha S, Lal M, Mir M H (2012). RSC Adv.

[8] Saha B, Frett B, Wang Y, Li H-Y (2013). Tetrahedron Lett.

[9] Brandi A, Cicchi S, Cordero F M (2008). Chem Rev.

[10] Tejedor D, García-Tellado F (2007). Chem Soc Rev.

[R11] 11CCDC 963354 contains the supplementary crystallographic data of product **3a**. These data can be obtained free of charge from The Cambridge Crystallographic Data Centre via http://www.ccdc.cam.ac.uk/data_request/cif.

[12] Bez G, Zhao C-G (2003). Org Lett.

[13] Oshita M, Yamashita K, Tobisu M, Chatani N (2005). J Am Chem Soc.

[14] Korotkov V S, Larionov O V, de Meijere A (2006). Synthesis.

[15] Masdeu C, Gómez E, Williams N A O, Lavilla R (2007). Angew Chem, Int Ed.

[16] Baldwin J E (1976). J Chem Soc, Chem Commun.

[17] Alabugin I V, Gilmore K, Manoharan M (2011). J Am Chem Soc.

[18] Nguyen M T, Hegarty A F, Sana M, Leroy G (1985). J Am Chem Soc.

[19] Johnson J E, Cornell S C (1980). J Org Chem.

[20] Tumanov V V, Tishkov A A, Mayr H (2007). Angew Chem, Int Ed.

[21] Schneekloth J S, Kim J, Sorensen E J (2009). Tetrahedron.

[22] Burkhard J A, Wagner B, Fischer H, Schuler F, Müller K, Carreira E M (2010). Angew Chem, Int Ed.

[23] Lowe J T, Lee M D, Akella L B, Davoine E, Donckele E J, Durak L, Duvall J R, Gerard B, Holson E B, Joliton A (2012). J Org Chem.

