# Supplementary material for: Studies on the interaction of isocyanides with imines: reaction scope and mechanistic variations
Source: Beilstein J Org Chem. 2014 Jan 6;10:12–7. doi: 10.3762/bjoc.10.3 (PMC3896293; doi:10.3762/bjoc.10.3)

Supporting Information

for

Studies on the interaction of isocyanides with imines: reaction scope and mechanistic variations

Ouldouz Ghashghaei,[1] Consiglia Annamaria Manna,[1] Esther Vicente-García,[1] Marc Revés,[1] and Rodolfo Lavilla*[1,2]

Address: [1]Barcelona Science Park, University of Barcelona, Baldiri Reixac 10–12, 08028 Barcelona, Spain and [2]Laboratory of Organic Chemistry, Faculty of Pharmacy, University of Barcelona, Av. Joan XXIII sn, 08028 Barcelona, Spain

Email: Rodolfo Lavilla - rlavilla@pcb.ub.es

* Corresponding author

Experimental procedures, characterization data, copies of the NMR spectra for all new compounds and X-ray views of azetidine 3a

Content

Experimental procedures	S3
Characterization data	S5
Copies of the NMR spectra	S16
X-ray views of azetidine **3a**	S34

Experimental procedures

General information

Unless stated otherwise, all reactions were carried out under argon atmosphere in dried glassware. Commercially available reactants were used without further purification. Thin-layer chromatography was performed on pre-coated Merk silica gel 60 F254 plates and visualized under an UV lamp. ^{1}H, and ^{13}C NMR spectra were recorded on a Varian Mercury 400 (at 400 MHz, and 100 MHz respectively). Unless otherwise quoted, NMR spectra were recorded in $CDCl_3$ solution with TMS as an internal reference. Data for ^{1}H NMR spectra are reported as follows: chemical shift (δ ppm), multiplicity, integration and coupling constants (Hz). Data for ^{13}C NMR spectra are reported in terms of chemical shift (δ ppm). IR spectra were recorded using a Thermo Nicolet Nexus spectrometer and are reported in frequency of absortion (cm^{-1}). High resolution mass spectrometry was performed by the University of Barcelona Mass Spectrometry Service.

The microwave-assisted reactions were performed by setting the temperature at reported number and the maximum power and pressure at 250 W and 200 psi respectively. The power is automatically altered by the device to maintain the set temperature for the reported duration of time. A ramp period of 10 min is set to reach the desired temperature and the device automatically starts the emission period as soon as the set temperature is obtained. During the reactions the stirring option is on and a stream of nitrogen cools the reactor. The Max Power option is also on to maintain the reaction temperature while cooling. This option should not be used for polar solvents.

The *N*-aryl-aromatic imines **1** were prepared by mixing the amine and carbonyl components in ACN, in the presence of 4 Å MS at room temperature, according to a standard method. *N*-alkyl imines and activated substrates were prepared following the described procedures (see below).

Preparation of imines and related compounds (1)

General procedure

Equimolar amounts of the corresponding aldehyde and amine (1:1, 2 mmol) together with 2 g of activated MS 4 Å were placed in a 50 mL flask, 10 mL of dry ACN were added and the solution was stirred under nitrogen for 24 h or until all the starting material was consumed. The reaction progress was monitored by TLC or HPLC. After the reaction was complete, 10 mL of saturated aqueous $NaHCO_3$ solution were added. Molecular sieves were filtered and washed with 20 mL of EtOAc. The aqueous layer was extracted with 3 × 10 mL of EtOAc. The combined organic layers were dried over Na_2SO_4, filtered and concentrated in vacuo. The resulting crude imine was used without further purification.

General procedure for isatin imines

Equimolar amounts of isatin and the corresponding amine (1:1, 5 mmol) were placed in a 100 mL flask, 20 mL of absolute EtOH and 0.2 mL glacial acetic acid were added and the mixture was refluxed for 12 h or until all the starting material was consumed. The reaction progress was monitored by TLC or HPLC. After the reaction was complete, the solution was set aside to gradually cool down overnight. The formed crystals were filtered, washed with cold EtOH and dried in vacuo [1].

The following compounds were prepared according to literature procedures:

N-(4-Chlorobenzylidene)propan-1-amine [2]

(E)-Ethyl 2-((4-methoxyphenyl)imino)acetate [3]

(S)-(+)-N-(4-Chlorobenzylidene)-4-methylbenzenesulfinamide [4]

1-(4-Chlorobenzylidene)-2-(4-methoxyphenyl)hydrazine [5]

4-Chlorobenzaldehyde oxime [6]

General procedures for the azetidine formation

General procedure A

0.5 mmol (1 equiv) of imine **1** were dissolved in 2 mL of dry THF. To this solution was dropwise syringed 0.5 mmol (1 equiv) of $BF_3 \cdot Et_2O$ while stirring. In case of using a solid isocyanide, it was first dissolved in 1 mL of THF. After 2 min, 1.5 mmol (3 equiv.) of the corresponding isocyanide was gradually added. The solution was stirred for 24 h or until the reaction was complete or showed no evolution. The reaction was quenched with 10 mL of saturated $NaHCO_3$ solution and extracted with 3 × 10 mL of AcOEt. The combined organic phases were dried over Na_2SO_4, concentrated in vacuo and purified by flash chromatography (Hexanes/EtOAc).

General procedure B

In a microwave reactor tube, 0.5 mmol (1 equiv) of imine **1** were dissolved in 2 mL of dry THF .To this solution was dropwise syringed 0.5 mmol (1 equiv) of $BF_3 \cdot Et_2O$ while stirring. After 2 minutes, 1.5 mmol (3 equiv) of the corresponding isocyanide was gradually added. In case of using a solid isocyanide, it was first dissolved in 1 mL of THF. The solution was irradiated in the microwave for 30 min at 65 °C. Then it was allowed to cool to room temperature. The reaction was quenched with 10 mL of saturated $NaHCO_3$ solution and extracted with 3 × 10 mL of EtOAc. The combined organic phases were dried over Na_2SO_4, concentrated in vacuo and purified by flash chromatography (hexanes/EtOAc).

Characterization data

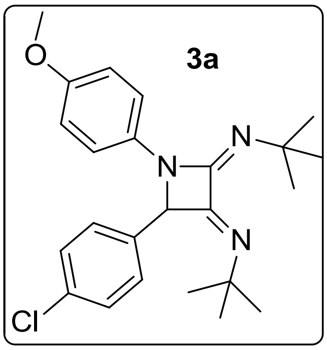

N,N'-(4-(4-Chlorophenyl)-1-(4-methoxyphenyl)azetidine-2,3-diylidene)bis(2-methylpropan-2-amine) **(3a).**

According to procedure B, azetidine **3a** was obtained as a pale yellow solid (48%).

$C_{24}H_{30}ClN_3O$ (M = 411.97 g·mol^{-1}).

IR (neat, cm^{-1}): 3417, 2969, 2930, 2860, 1719, 1668, 1508, 1380, 1303, 1245, 1207, 1130, 1028, 932, 829.

^{1}H NMR (400 MHz, $CDCl_3$) δ 7.42 (d, *J* = 9.1 Hz, 2H), 7.33 (s, 4H), 6.76 (d, *J* = 9.1 Hz, 2H), 5.38 (s, 1H), 3.71 (s, 3H), 1.49 (s, 9H), 1.09 (s, 9H).

^{13}C NMR (101 MHz, $CDCl_3$) δ 154.6, 153.9, 152.3, 136. 8, 134.4, 134.0, 129.36, 129.35, 117.2, 114.2, 70.3, 58.1, 55.5, 53.9, 53.5, 30.8, 30.2.

HRMS (ESI): calculated for **$C_{24}H_{31}ClN_3O$** $[M + H]^+$: 412.215, found 412.215.

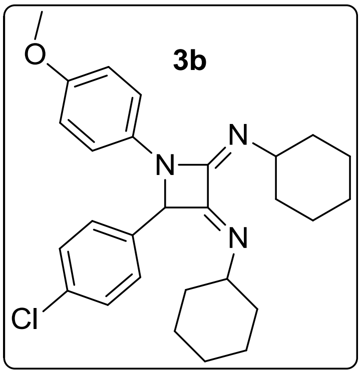

***N,N'-(4-(4-Chlorophenyl)-1-(4-methoxyphenyl) azetidine-2,3-diylidene)dicyclohexanamine* (3b).**

According to procedure B, using 20% $BF_3\cdot Et_2O$, **3b** was obtained as an amorphous solid (41%).

$C_{28}H_{34}ClN_3O$ (M = 464.04 g·mol^{-1}).

IR (neat, cm^{-1}): 2930, 2853, 1713, 1674, 1508, 1444, 1367, 1297, 1245, 1137, 1008, 823, 733.

^{1}H NMR (400 MHz, $CDCl_3$) δ 7.29 (d, *J* = 9.1 Hz, 2H), 7.25 (d, *J* = 9.6 Hz, 2H), 7.20 (d, *J* = 9.6 Hz, 2H), 6.69 (d, *J* = 9.1 Hz, 2H), 5.39 (s, 1H), 4.21 (m, 2H), 3.64 (s, 3H), 3.18 (m, 4H), 1.81 – 0.75 (m, 16H).

^{13}C NMR (101 MHz, $CDCl_3$) δ 157.1, 154.5, 152.2, 135.6, 134.1, 133.8, 129.3, 128.2, 116.9, 114.3, 69.3, 61.2, 57.1, 55.49, 55.44, 35.34, 35.26, 33.6, 32.8, 25.9, 25.5, 24.8, 23.87, 23.72.

HRMS (ESI): calculated for **$C_{28}H_{35}ClN_3O$** $[M + H]^+$ 464.2463, found 464.2452.

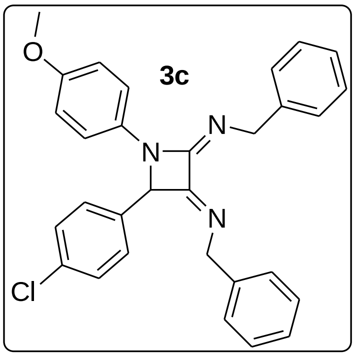

***2,3-Bis(benzylimino)-4-(4-chlorophenyl)-1-(4-methoxy-phenyl)azetidine* (3c).**

According to procedure A, using 20% $BF_3\cdot Et_2O$**,** azetidine **3c** was obtained as an amorphous solid (63%).

$C_{30}H_{26}ClN_3O$ (M = 480.00 g·mol^{-1}).

IR (neat, cm^{-1}): 3058, 3026, 2930, 1726, 1687, 1508, 1450, 1245, 1130, 1028, 829.

^{1}H NMR (400 MHz, $CDCl_3$) 7.41 (ddd, *J* = 6.2, 1.3, 0.7 Hz, 2H), 7.35 – 7.31 (m, 2H), 7.28 (cs, 6H), 7.21 – 7.12 (m, 4H), 6.96 (dd, *J* = 7.9, 1.6 Hz, 2H), 6.70 (d, *J* = 9.1 Hz, 2H), 5.51 (s, 1H), 5.09 (s, 2H), 4.45 (d, *J* = 14.4 Hz, 1H), 4.41 (d, *J* = 14.4 Hz, 1H), 3.64 (s, 3H).

^{13}C NMR (101 MHz, $CDCl_3$) δ 160.0, 154.0, 152.3, 140.7, 136.7, 133.8, 133.2, 132.1, 128.6, 127.6, 127.4, 127.2, 126.6, 126.47, 126.1, 125.3, 116.2, 113.3, 68.9, 55.3, 54.4, 52.4.

HRMS (ESI) : calculated for $C_{30}H_{27}ClN_3O$ [M + H]$^+$ 480.1837., found 480.1835.

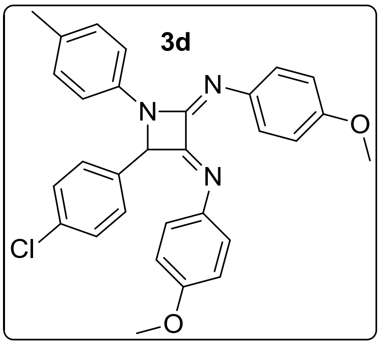

***N,N'-(4-(4-Chlorophenyl)-1-(p-tolyl)azetidine-2,3-diylidene)bis(4-methoxyaniline)* (3d)**

According to procedure B, azetidine **3d** was obtained as a yellow amorphous solid (19%).

$C_{30}H_{26}ClN_3O_2$ (M = 495.99 g·mol^{-1}).

IR (neat, cm^{-1}): 1656, 1504, 1499, 1378, 1294, 1241, 1030, 830.

^{1}H NMR (400 MHz, $CDCl_3$) 7.54 (d, *J* = 8.9 Hz, 2H), 7.44 (d, *J* = 8.5 Hz, 2H), 7.15 (d, *J* = 8.5 Hz, 2H), 7.07 (d, *J* = 8.0 Hz, 4H), 6.89 (d, *J* = 8.9 Hz, 2H), 6.74 (bs, 4H), 5.84 (s, 1H), 3.81 (s, 3H), 3.78 (s, 3H), 2.28 (s, 3H).

^{13}C NMR (101 MHz, $CDCl_3$) δ 159.1, 158.1, 156.6, 150.2, 139.71, 139.58, 136.8, 134.4, 133.1, 132.8, 129.7, 129.1, 128.4, 124.9, 122.6, 116.7, 114.05, 113.87, 69.3, 55.65, 55.58, 21.1.

HRMS (ESI): calculated for $C_{30}H_{27}ClN_3O_2$ [M + H]$^+$ 496.1786., found 496.1787.

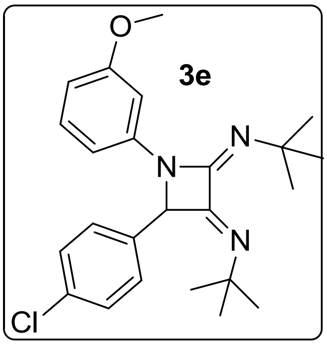

***N,N'-(4-(4-Chlorophenyl)-1-(3-methoxyphenyl)azetidine-2,3-diylidene)bis(2-methylpropan-2-amine)* (3e)**

According to procedure B, azetidine **3e** was obtained as a cream amorphous solid (19%).

$C_{24}H_{30}ClN_3O$ (M = 411.97 g·mol^{-1}).

IR (neat, cm^{-1}): 2962, 2861, 1722, 1671, 1477, 1203, 837.

^{1}H NMR (400 MHz, $CDCl_3$) δ 7.51 (t, *J* = 2.1 Hz, 1H), 7.34 (bs, 4H), 7.04 (t, *J* = 8.2 Hz, 1H), 6.72 (dd, *J* = 8.2, 1.2 Hz, 1H), 6.45 (ddd, *J* = 8.2, 2.1, 1.2 Hz, 1H), 5.39 (s, 1H), 3.74 (s, 3H), 1.48 (s, 9H), 1.09 (s, 9H).

^{13}C NMR (101 MHz, $CDCl_3$) δ 159.9, 153.6, 152.3, 141.1, 136.5, 134.4, 129.32, 129.26, 129.20, 107.90, 107.81, 101.9, 70.4, 58.1, 55.0, 53.9, 30.6, 30.0.

HRMS (ESI): calculated for **$C_{24}H_{31}ClN_3O$** [M + H]$^+$: 412.215, found 412.215.

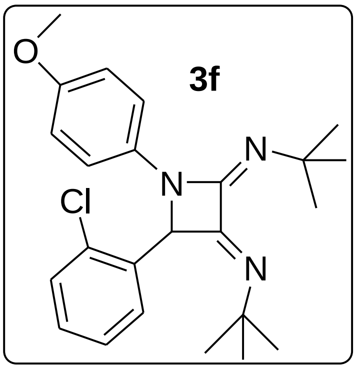

***N,N'-(4-(2-Chlorophenyl)-1-(4-methoxyphenyl)-azetidine-2,3-diylidene)bis(2-methylpropan-2-amine)* (3f)**

According to procedure B, azetidine **3f** was obtained as a cream solid (12%).

$C_{24}H_{30}ClN_3O$ (M = 411.97 g·mol^{-1}).

IR (neat, cm^{-1}): 3084, 2952, 2819, 1721, 1660, 1507, 1233, 1121, 754.

^{1}H NMR (400 MHz, $CDCl_3$) δ 7.47 (d, *J* = 9.0 Hz, 2H), 7.40 (dd, *J* = 6.9, 2.9 Hz, 1H), 7.35 (dd, *J* = 6.9, 3.6 Hz, 1H), 7.21 (m, 2H), 6.77 (d, *J* = 9.0 Hz, 2H), 5.99 (s, 1H), 3.71 (s, 3H), 1.48 (s, 9H), 1.09 (s, 9H).

^{13}C NMR (101 MHz, $CDCl_3$) δ 154.7, 154.4, 153.4, 152.5, 135.8, 133.66, 133.56, 131.8, 129.89, 129.79, 129.69, 128.9, 127.8, 122.5, 117.1, 114.2, 65.7, 57.6, 55.4, 53.7, 30.7, 29.6.

HRMS (ESI): calculated for **$C_{24}H_{31}ClN_3O$** [M + H]$^+$: 412.215, found 412.215.

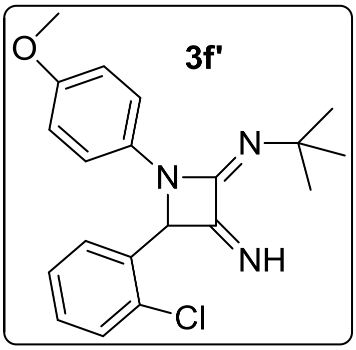

***N*-(4-(2-Chlorophenyl)-3-imino-1-(4-methoxyphenyl)-azetidin-2-ylidene)-2-methylpropan-2-amine** **(3f')**

According to procedure B, azetidine **3f´** was obtained as yellow oil (15%).

$C_{20}H_{22}ClN_3O$ (M = 355.86 g·mol^{-1}).

IR (neat, cm^{-1}): 3379, 2962, 2820, 1640, 1508, 1233, 1040, 806.

^{1}H NMR (400 MHz, $CDCl_3$) δ 7.47 – 7.43 (m, 1H), 7.37 – 7.33 (m, 1H), 7.31 – 7.14 (m, 2H), 6.73 (d, *J* = 9.0 Hz, 2H), 6.59 (d, *J* = 9.0 Hz, 2H), 5.68 (d, *J* = 5.4 Hz, 1H), 5.25 (d, *J* = 5.4 Hz, 1H), 3.71 (s, 3H), 1.43 (s, 9H).

^{13}C NMR (101 MHz, $CDCl_3$) δ 152.7, 139.7, 137.8, 135.2, 134.6, 130.2, 129.8, 128.8, 127.7, 115.1, 114.8, 111.2, 61.3, 58.7, 55.7, 29.3.

HRMS (ESI): calculated for **$C_{20}H_{23}ClN_3O$** [M + H]$^+$: 356.1524, found 356.1513.

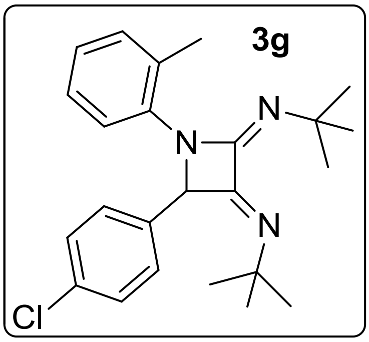

***N,N'*-(4-(4-Chlorophenyl)-1-(o-tolyl)azetidine-2,3-diylidene)bis(2-methylpropan-2-amine)** **(3g)**

According to procedure B, azetidine **3g** was obtained as a brown paste (31%).

$C_{24}H_{30}ClN_3$ (M = 395.97 g·mol^{-1}).

IR (neat, cm^{-1}): 2952, 2861, 2850, 1671, 1488, 1203, 837.

^{1}H NMR (400 MHz, $CDCl_3$) δ 7.45 (d, *J* = 8.1 Hz, 1H), 7.25 (d, *J* = 8.7 Hz, 2H), 7.20 (d, *J* = 8.7 Hz, 2H), 7.04 (m, 2H), 6.94 (m, 1H), 5.56 (s, 1H), 2.29 (s, 3H), 1.47 (s, 9H), 1.11 (s, 9H).

^{13}C NMR (101 MHz, $CDCl_3$) δ 154.9, 153.0, 137.0, 136.4, 134.2, 132.0, 131.2, 129.4, 129.0, 125.9, 125.0, 122.8, 118.6, 114.9, 71.6, 58.2, 53.6, 30.7, 30.0, 20.1.

HRMS (ESI): calculated for **$C_{24}H_{31}ClN_3$** $[M + H]^+$: 396.2201, found 396.2194.

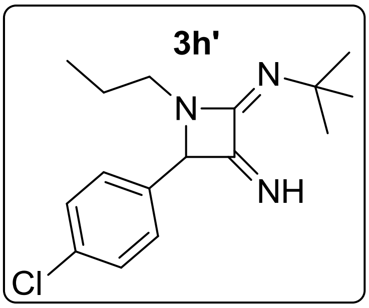

***N-(4-(4-Chlorophenyl)-3-imino-1-propylazetidin-2-ylidene)-2-methylpropan-2-amine* (3h')**

According to procedure B, azetidine **3h´** was obtained as a lemon amorphous solid (32%).

$C_{16}H_{22}ClN_3$ (M = 291.82 g·mol^{-1}).

IR (neat, cm^{-1}): 3359, 3318, 2952, 2861, 2149, 1579, 1091, 837.

^{1}H NMR (400 MHz, $CDCl_3$) δ 7.42 (d, *J* = 8.4 Hz, 2H), 7.32 (d, *J* = 8.4 Hz, 2H), 5.79 (s, 1H), 2.75 (cs, 2H), 1.57 (s, 1H), 1.41 (cs, 2H), 1.17 (s, 9H), 0.76 (t, *J* = 7.4 Hz, 3H).

^{13}C NMR (101 MHz, $CDCl_3$) δ 161.1, 136.0, 131.1, 130.5, 129.1, 123.4, 54.7, 46.1, 29.6, 29.1, 24.5, 11.3.

HRMS (ESI): calculated for **$C_{16}H_{23}ClN_3$** $[M + H]^+$: 292.1575, found 292.1575.

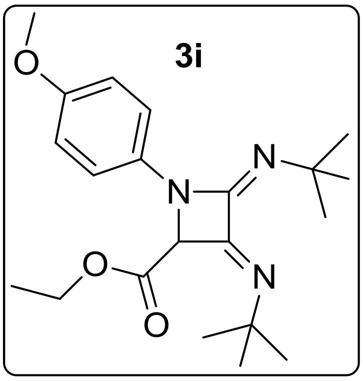

***Ethyl 3,4-bis(tert-butylimino)-1-(4-methoxyphenyl) azetidine-2-carboxylate* (3i)**

According to procedure A, using 20% BF_3, Et_2O, azetidine **3i** was obtained as a white amorphous solid (9%).

$C_{21}H_{31}N_3O_3$ (M = 373.49 g·mol^{-1}).

IR (neat, cm^{-1}): 2960, 1737, 1717, 1674, 1187.

^{1}**H NMR** (400 MHz, $CDCl_3$) δ 7.46 (d, *J* = 9.1 Hz, 2H), 6.85 (d, *J* = 9.1 Hz, 2H), 4.95 (s, 1H), 4.19 (cs, 2H), 3.77 (s, 3H), 1.42 (s, 9H), 1.30 (s, 9H), 1.20 (t, *J* = 7.1 Hz, 3H).

^{13}**C NMR** (126 MHz, $CDCl_3$) δ 169.6, 154.8, 151.4, 147.7, 134.2, 116.7, 114.5, 68.5, 62.0, 58.5, 55.7, 54.0, 30.7, 29.9, 14.1.

HRMS (ESI): calculated for $\mathbf{C_{21}H_{32}N_3O_3}$ $[M + H]^+$: 374.2438, found 374.2441.

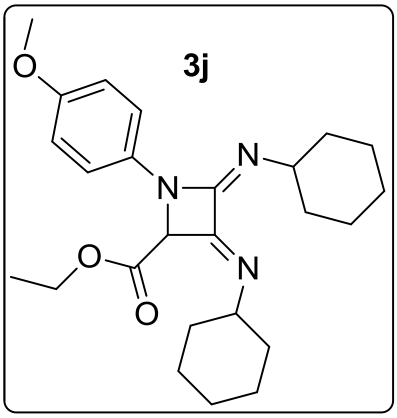

Ethyl 3,4-bis(cyclohexylimino)-1-(4-methoxyphenyl)azetidine-2-carboxylate **(3j)**

According to procedure A, using 20% $BF_3{\cdot}Et_2O$, azetidine **3j** was obtained as an amorphous white solid (34%).

$\mathbf{C_{25}H_{35}N_3O_3}$ (M = 425.56 g·mol^{-1}).

IR (neat, cm^{-1}): 2930, 2854, 1720, 1687, 1514.

^{1}**H NMR** (400 MHz, $CDCl_3$) δ 7.27 (d, *J* = 8.0, Hz, 2H), 6.79 (d, *J* = 8.0 Hz, 2H), 4.98 (s, 1H), 4.16 (m, Hz, 2H), 4.07 (m, 2H), 3.70 (s, 3H), 3.38 (m, 4H), 1.72 - 1.25(m, 16H), 1.18 (t, *J* = 7,0 Hz, 3H).

^{13}**C NMR** (101 MHz, $CDCl_3$) δ 206.5, 154.5, 134.0, 116.5, 114.4, 67.0, 62.00, 61.98, 61.9, 57.3, 55.5, 35.1, 33.6, 33.4, 30.9, 25.8, 25.5, 24.6, 23.8, 23.7, 14.1 (three quaternary carbon signals not detected)

HRMS (ESI): calculated for $\mathbf{C_{25}H_{30}N_4O}$ $[M + H]^+$: 426.2751, found 426.2745.

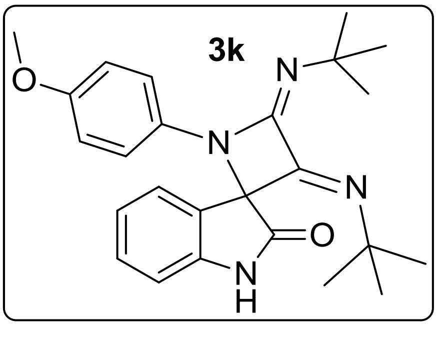

3,4-bis(tert-Butylimino)-1-(4-methoxyphenyl) spiro[azetidine-2,3'-indolin]-2'-one **(3k)**

According to procedure B, azetidine **3k** was obtained as a brown paste (14%).

$C_{25}H_{30}N_4O_2$ (M = 418.53 g·mol^{-1}).

^{1}H NMR (400 MHz, $CDCl_3$) δ 9.05 (bs, 1H), 7.45 (m, 1H), 7.35 (m, 1H), 7.31 – 7.14 (m, 2H), 6.73 (d, *J* = 9.0 Hz, 2H), 6.59 (d, *J* = 9.0 Hz, 2H), 3.71 (s, 3H), 1.43 (s, 9H), 0.98 (s, 9H).

LRMS (HPLC-MS): calculated for **$C_{25}H_{31}N_4O_2$** [M + H]$^+$: 419.24, found 419.

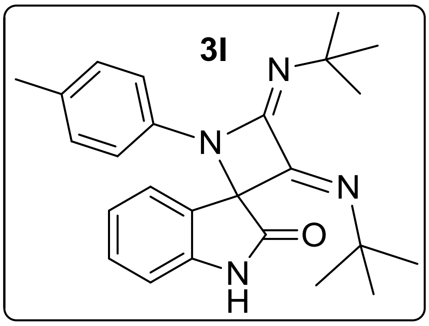

***3,4-bis(tert-Butylimino)-1-(p-tolyl)spiro[azetidine-2,3'-indolin]-2'-one* (3l)**

According to procedure A, azetidine **3l** was obtained as an amorphous cream solid (28%).

$C_{25}H_{30}N_4O$ (M = 402.53 g·mol^{-1}).

IR (neat, cm^{-1}): 2962, 2861, 1742, 1691, 1508, 1355, 1183, 928.

^{1}H NMR (400 MHz, $CDCl_3$) δ 9.42 (s, 1H), 7.27 (m, *J* = 7.8, 1.2 Hz, 1H), 7.22 (cs, 3H), 7.01 (m, *J* = 7.8, 0.8 Hz, 1H), 6.93 – 6.90 (m, 2H), 6.89 (s, 1H), 2.17 (s, 3H), 1.50 (s, 9H), 1.02 (s, 9H).

^{13}C NMR (101 MHz, $CDCl_3$) δ 175.4, 150.7, 147.3, 140.4, 136.9, 131.6, 130.5, 129.4, 125.44, 124.5, 123.5, 116.0, 111.3, 77.4, 58.5, 54.1, 30.6, 29.5, 20.8.

HRMS (ESI): calculated for **$C_{25}H_{31}N_4O$** [M + H]$^+$: 403.2492, found 403.249.

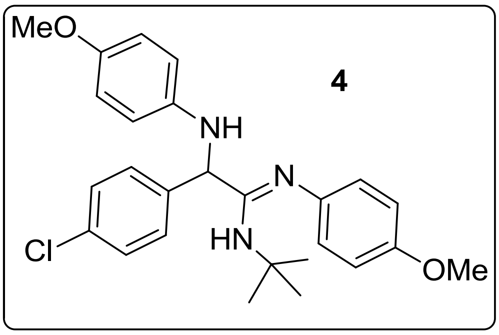

***N-(tert-Butyl)-2-(4-chlorophenyl)-N'-(4-methoxyphenyl)-2-((4-methoxyphenyl) amino)acetimidamide* (4)**

Amidine **4a** was obtained by direct stirring aniline **1a**, aldehyde **2a** and tert-butyl

isocyanide, with $Sc(OTf)_3$ (20% mol) at rt. for 14 h in ACN. After standard aqueous work up and EtOAc extraction, the crude was purified through column chromatography (SiO2, Hexanes-EtOAC 50:50) to yield amidine **4** (28%).

$C_{26}H_{30}ClN_3O_2$ (M = 451.99 g·mol^{-1}).

^{1}H NMR (400 MHz, $CDCl_3$) δ 7.25 (d, *J* = 8.5 Hz, 2H), 7.08 (d, *J* = 8.5 Hz, 2H), 6.83 (d, *J* = 9.0 Hz, 2H), 6.65 (cs, 4H), 6.40 (d, *J* = 8.8 Hz, 2H), 6.00 (m, 1H), 4.86 (s, 1H), 3.78 (s, 3H), 3.73 (s, 3H), 3.46 (bs, 1H), 1.46 (s, 9H).

^{13}C NMR (101 MHz, $CDCl_3$) δ 154.5, 153.4, 143.8, 141.0, 138.5, 134.0, 129.4, 128.9, 122.9, 116.4, 114.93, 114.80, 114.8, 113.96, 59.7, 55.7, 55.5, 50.8, 28.4.

LRMS (HPLC-MS): calculated for **$C_{26}H_{31}ClN_3O_2$** [M + H]$^+$: 452.20, found 452.

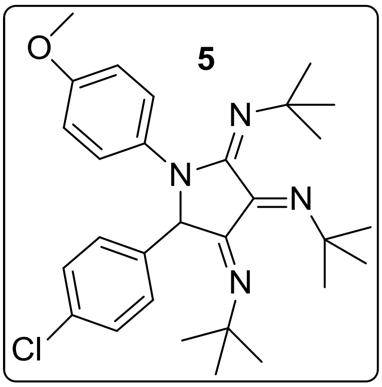

***N,N',N''-(5-(4-Chlorophenyl)-1-(4-methoxyphenyl)-pyrrolidine-2,3,4-triylidene)tris(2-methylpropan-2-amine)* (5)**

According to procedure B, pyrrolidine **5** was obtained as a brown paste (9%).

$C_{29}H_{39}ClN_4O$ (M = 495.10 g·mol^{-1}).

IR (neat, cm^{-1}): 2962, 1691, 1640, 1508, 1355, 1233, 816.

^{1}H NMR (400 MHz, $CDCl_3$) δ 7.32 (d, *J* = 9.2 Hz, 2H), 7.26 (d, *J* = 8.5 Hz, 2H), 7.16 (d, *J* = 8.5 Hz, 2H), 6.73 (d, *J* = 9.2 Hz, 2H), 5.59 (s, 1H), 3.74 (s, 3H), 1.45 (s, 9H), 1.43 (s, 9H), 1.29 (s, 9H).

HRMS (ESI): calculated for **$C_{29}H_{40}ClN_4O$** [M + H]$^+$: 495.2885, found 495.2875.

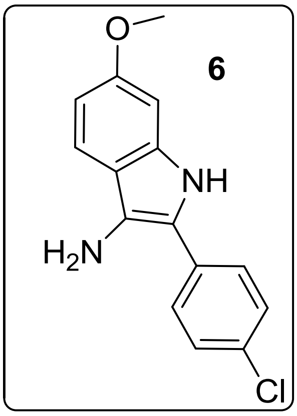

***2-(4-Chlorophenyl)-6-methoxy-1H-indol-3-amine* (6)**

According to procedure B, indole **6** was obtained as an slightly unstable amorphous cream solid (27%).

$C_{15}H_{13}ClN_2O$ (M = 272.72 g·mol^{-1}).

IR (neat, cm^{-1}): 3237, 2952, 2881, 2393, 2220, 1488, 1152, 1020, 827.

^{1}H NMR (400 MHz, DMSO) δ 12.45 (m, 1H), 7.96 (d, *J* = 8.6 Hz, 2H), 7.71 (d, *J* = 8.6 Hz, 2H), 7.54 (d, *J* = 8.7 Hz, 1H), 7.01 (d, *J* = 2.2 Hz, 1H), 6.93 (dd, *J* = 8.7, 2.2 Hz, 1H), 3.84 (s, 3H), 1.25 (s, 2H).

^{13}C NMR (101 MHz, DMSO) δ 157.8, 142.8, 137.0, 134.6, 129.8, 128.7, 122.5, 119.7, 112.9, 95.8, 55.9.

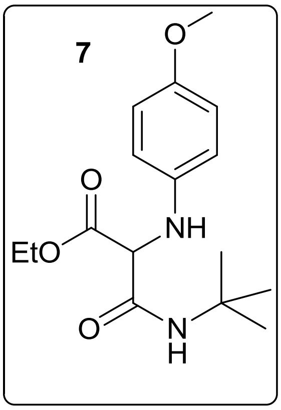

***Ethyl 3-(tert-butylamino)-2-(4-methoxyphenylamino)-3-oxopropanoate* (7)**

According to procedure A, aminoamide **7** was obtained as light brown paste (41%).

$C_{16}H_{24}N_2O_4$ (M = 308.37 g·mol^{-1}).

IR (neat, cm^{-1}): 3381, 2968, 1741, 1699, 1619, 1512, 1464, 1367, 1244, 1179, 1033.

^{1}H NMR (400 MHz, $CDCl_3$) δ 6.79 (d, *J* = 8.9 Hz, 2H), 6.64 (s, 1H), 6.60 (d, *J* = 8.9 Hz, 2H), 4.71 (s, 1H), 4.36 – 4.20 (m, 2H), 3.75 (s, 3H), 1.32 (s, 9H), 1.30 (t, *J* = 7.1 Hz, 3H).

^{13}C NMR (101 MHz, $CDCl_3$) δ 169.4, 165.5, 153.4, 139.6, 115.3, 114.8, 64.2, 62.4, 55.5, 51.3, 28.4, 14.0.

HRMS (ESI): calculated for $\mathbf{C_{16}H_{25}N_2O_4}$ [M + H]$^+$: 309.1809, found 309.1809.

8

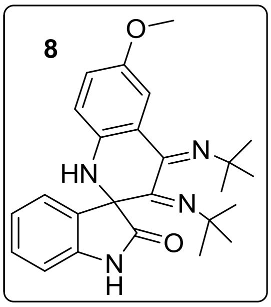

***3',4'-bis(tert-Butylimino)-6'-methoxy-3',4'-dihydro-1'H-spiro[indoline-3,2'-quinolin]-2-one* (8)**

According to procedure B, tetrahydroquinoline **8** was obtained as a brown solid (17%)

$\mathbf{C_{25}H_{30}N_4O_2}$ (M = 418.53 g·mol^{-1}).

IR (neat, cm^{-1}): 3420, 3227, 2962, 1681, 1508, 1223, 1040, 755.

^{1}H NMR (400 MHz, $CDCl_3$) δ 7.84 (dd, *J* = 7.8, 1.5 Hz, 1H), 7.70 (m, 1H), 7.42 (dd, *J* = 7.6, 1.3 Hz, 1H), 7.32 (m, *J* = 7.6, 1.3 Hz, 1H), 7.20 (bs, 2H), 7.12 (dd, *J* = 8.0, 1.0 Hz, 1H), 6.45 (bs, 1H), 5.27 (bs, 1H), 3.77 (s, 3H), 1.60 (s, 9H), 1.20 (s, 9H).

^{13}C NMR (101 MHz, $CDCl_3$) δ 167.2, 154.8, 153.4, 140.7, 130.3, 129.6, 128.4, 127.1, 125.7, 123.7, 121.5, 120.7, 119.6, 115.7, 105.7, 59.8, 55.6, 51.7, 29.7, 29.1.

HRMS (ESI): calculated for $\mathbf{C_{25}H_{31}N_4O_2}$ [M + H]$^+$: 419.2442, found 419.2438.

References

1. Kahveci, B. *Molecules* **2005,** *10*, 376-382.
2. Renault, S. V.; Bertrand, S.; Carreaux, F. O.; Bazureau, J. P. *Journal of Combinatorial Chemistry* **2007,** *9*, 935-942.
3. Gerard, B.; O'Shea, M. W.; Donckele, E.; Kesavan, S.; Akella, L. B.; Xu, H.; Jacobsen, E. N.; Marcaurelle, L. A. *ACS combinatorial science* **2012,** *14*, 621-630.
4. Davis, F. A.; Zhang, Y.; Andemichael, Y.; Fang, T.; Fanelli, D. L.; Zhang, H., *J. Org. Chem.* **1999**, *64*, 1403-1406.
5. Hania, M. M., *Journal of Chemistry* **2009,** *6*, S508-S514.
6. Galvis, C. E. P.; Kouznetsov, V. V. *Org. Biomol. Chem.* **2013,** *11*, 407-411.

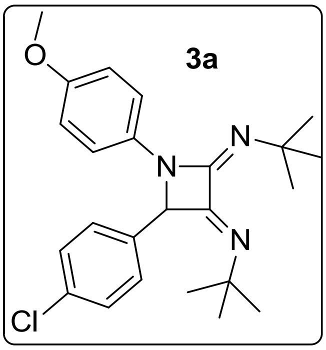

Copies of the NMR spectra

N,N'-(4-(4-Chlorophenyl)-1-(4-methoxyphenyl)azetidine-2,3-diylidene)bis(2-methylpropan-2-amine) (3a)

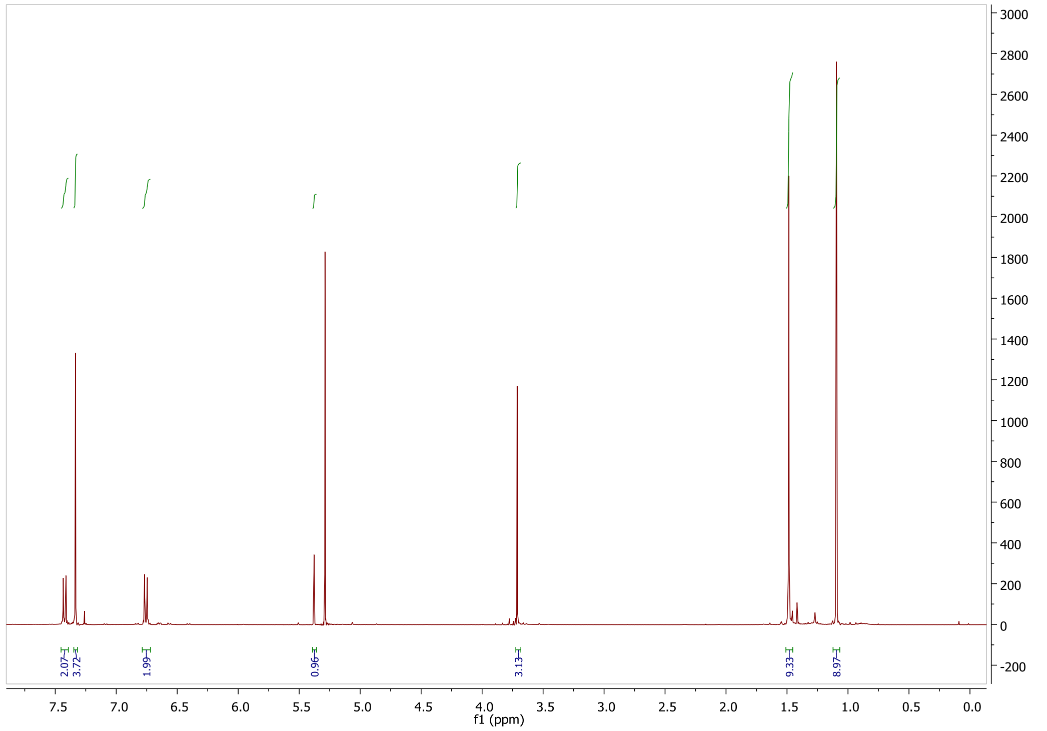

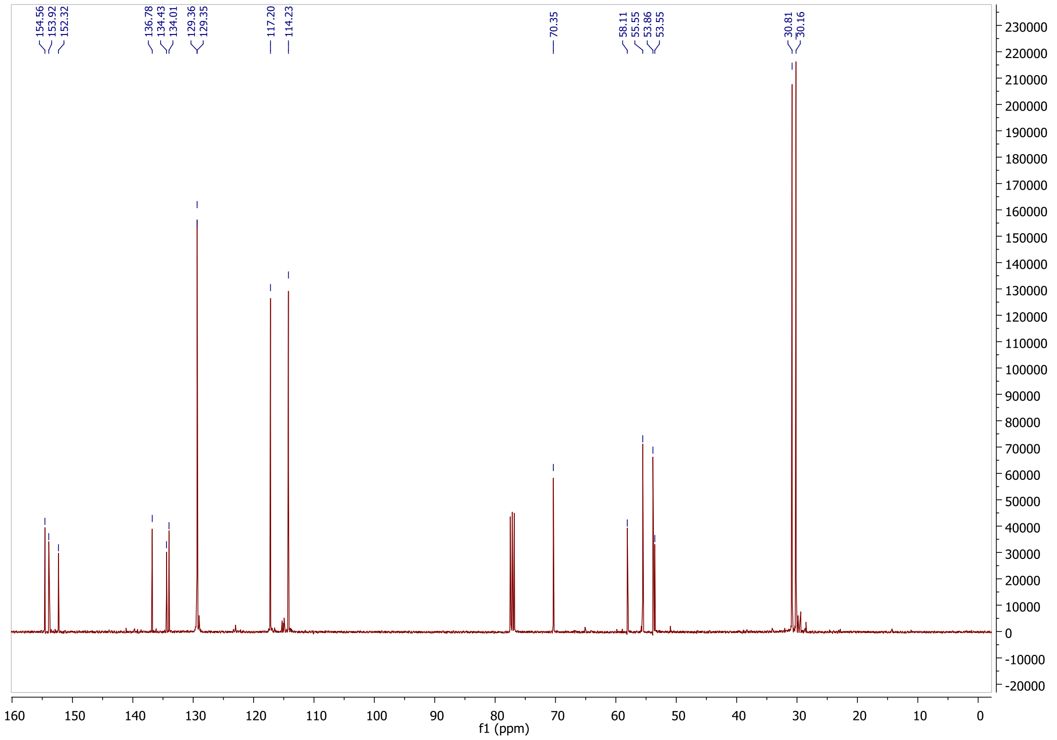

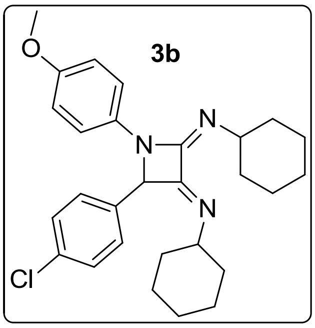

N,N'-(4-(4-Chlorophenyl)-1-(4-methoxyphenyl)azetidine-2,3-diylidene)dicyclohexanamine **(3b)**

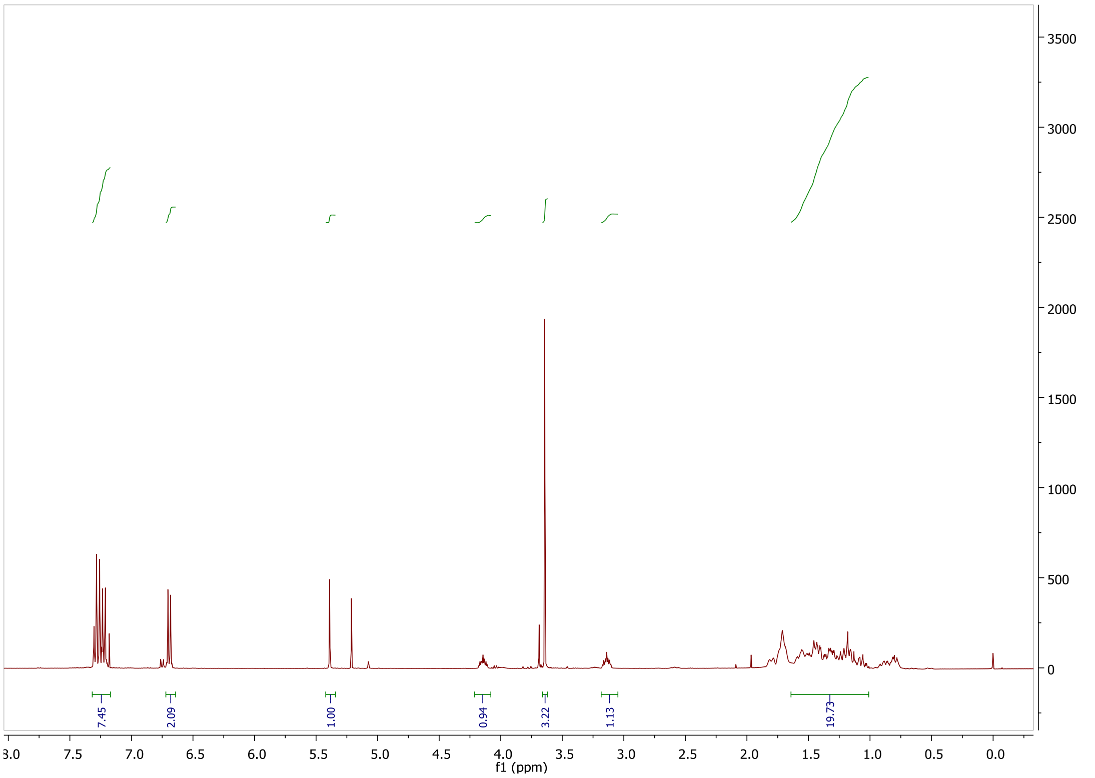

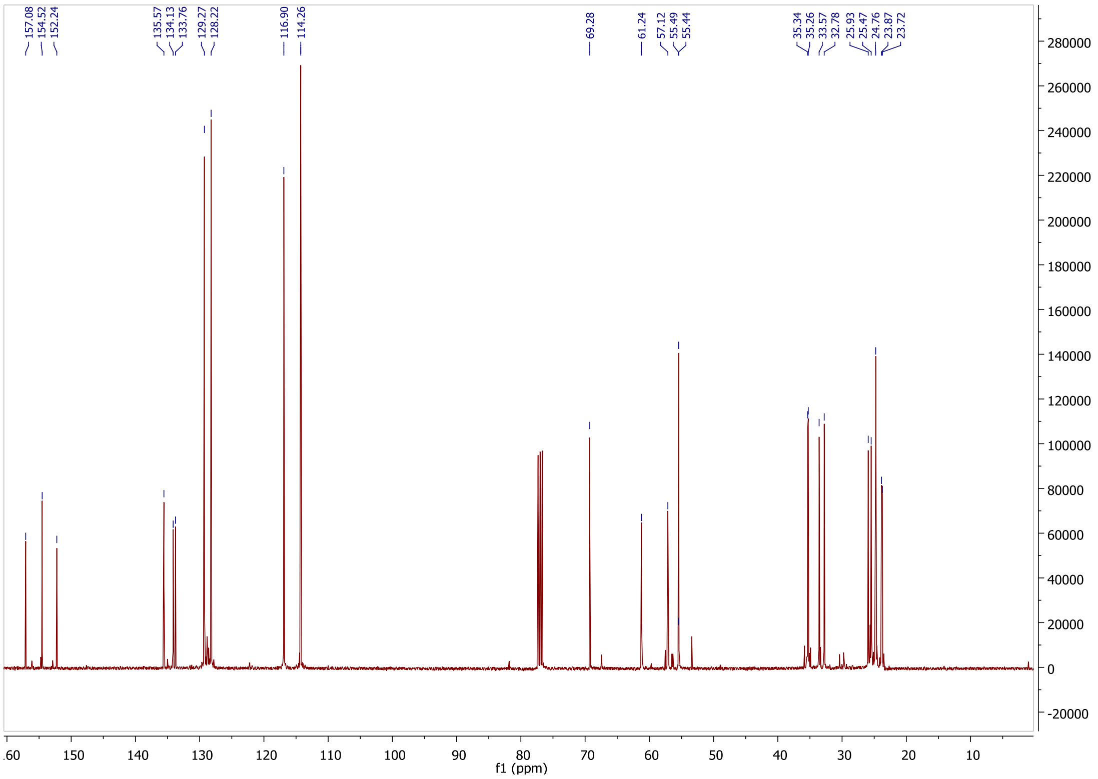

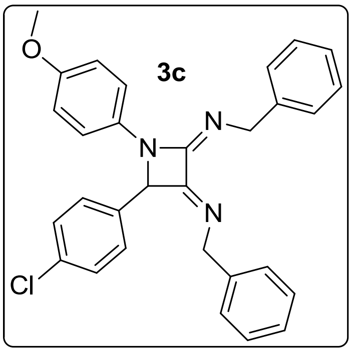

N,N'-(4-(4-Chlorophenyl)-1-(4-methoxyphenyl)azetidine-2,3-diylidene)bis(1-phenylmethanamine) **(3c)**

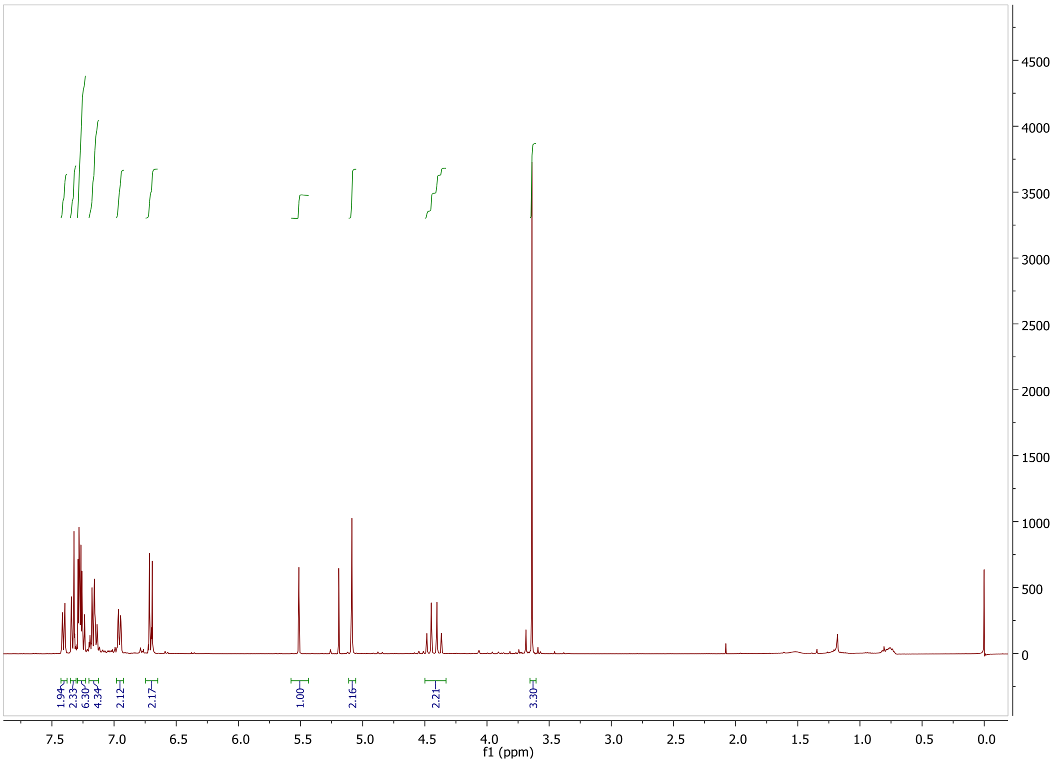

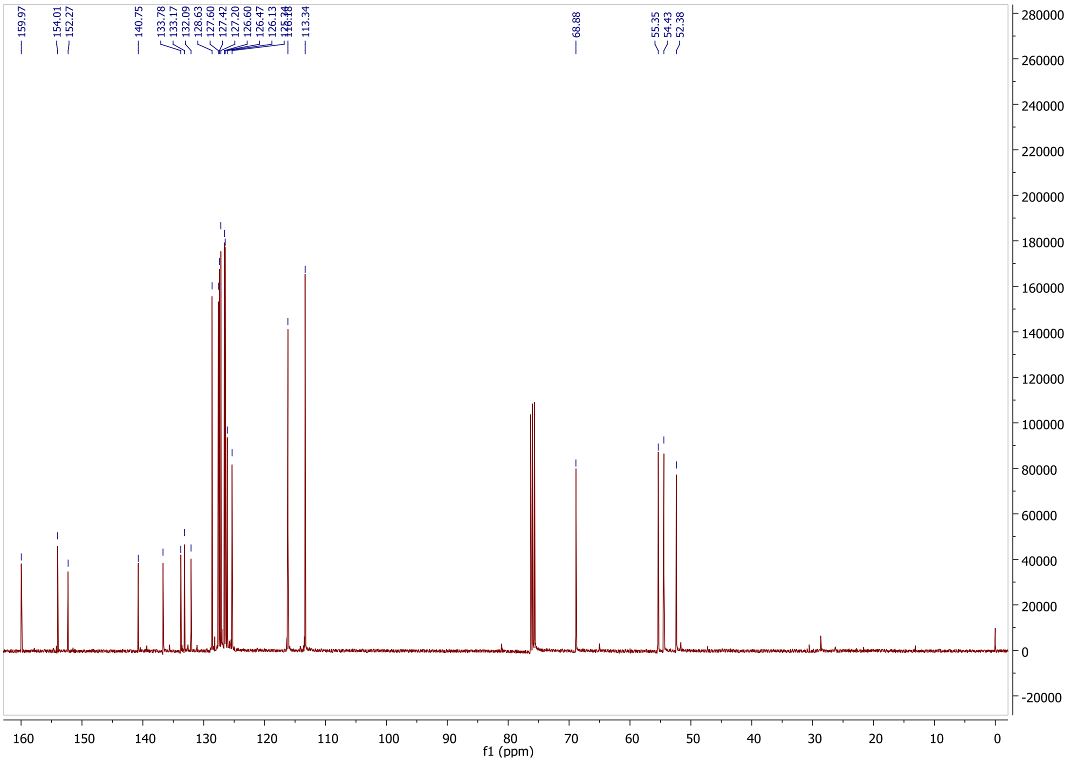

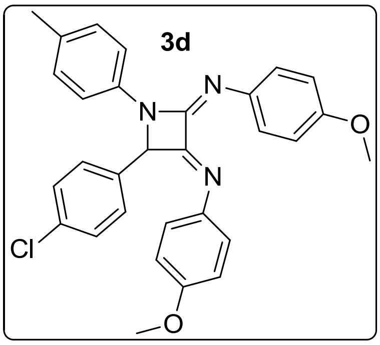

(N,N',N,N')-N,N'-(4-(4-Chlorophenyl)-1-(p-tolyl)azetidine-2,3-diylidene)bis(4-methoxyaniline) **(3d)**

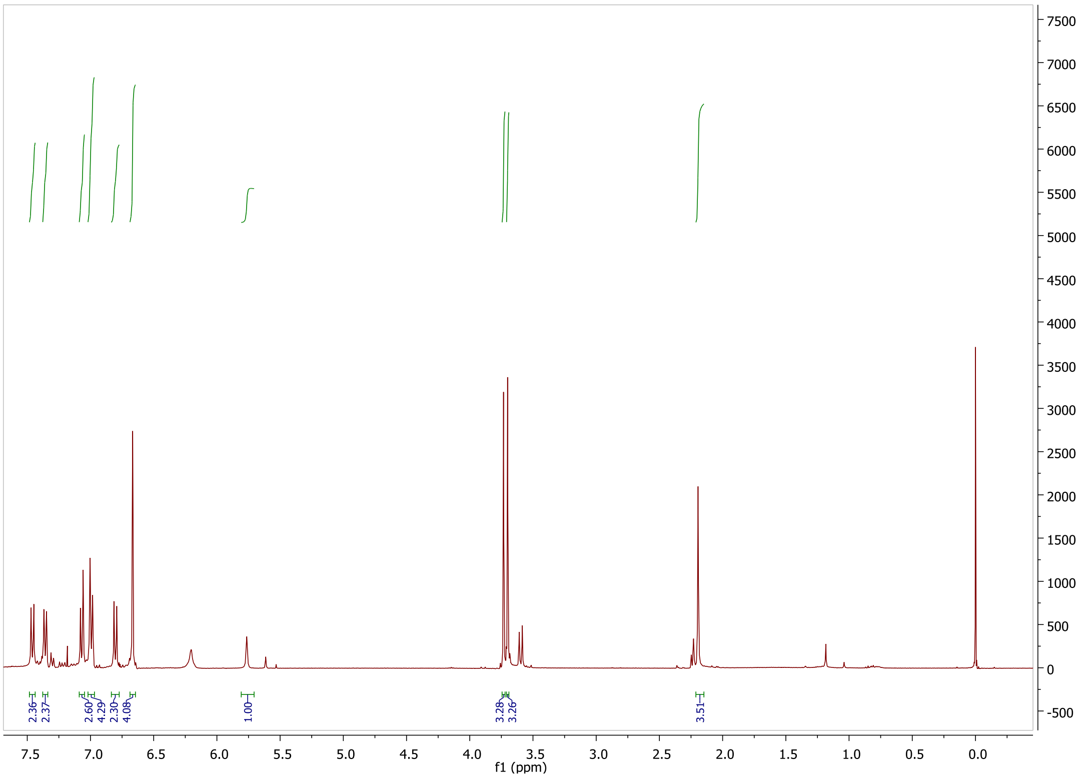

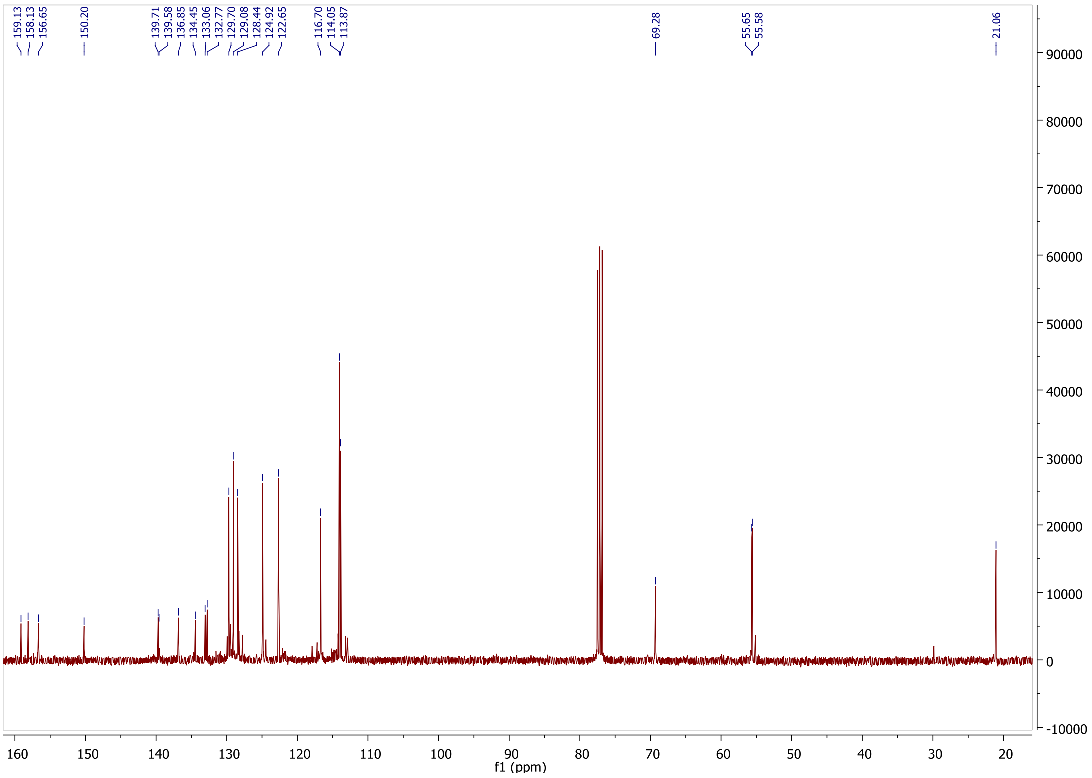

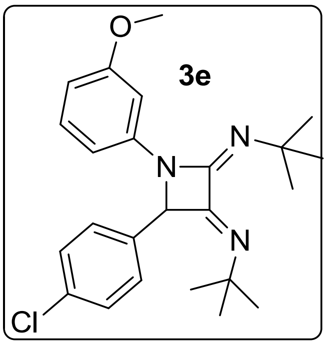

N,N'-(4-(4-Chlorophenyl)-1-(3-methoxyphenyl)azetidine-2,3-diylidene)bis(2-methylpropan-2-amine) (3e)

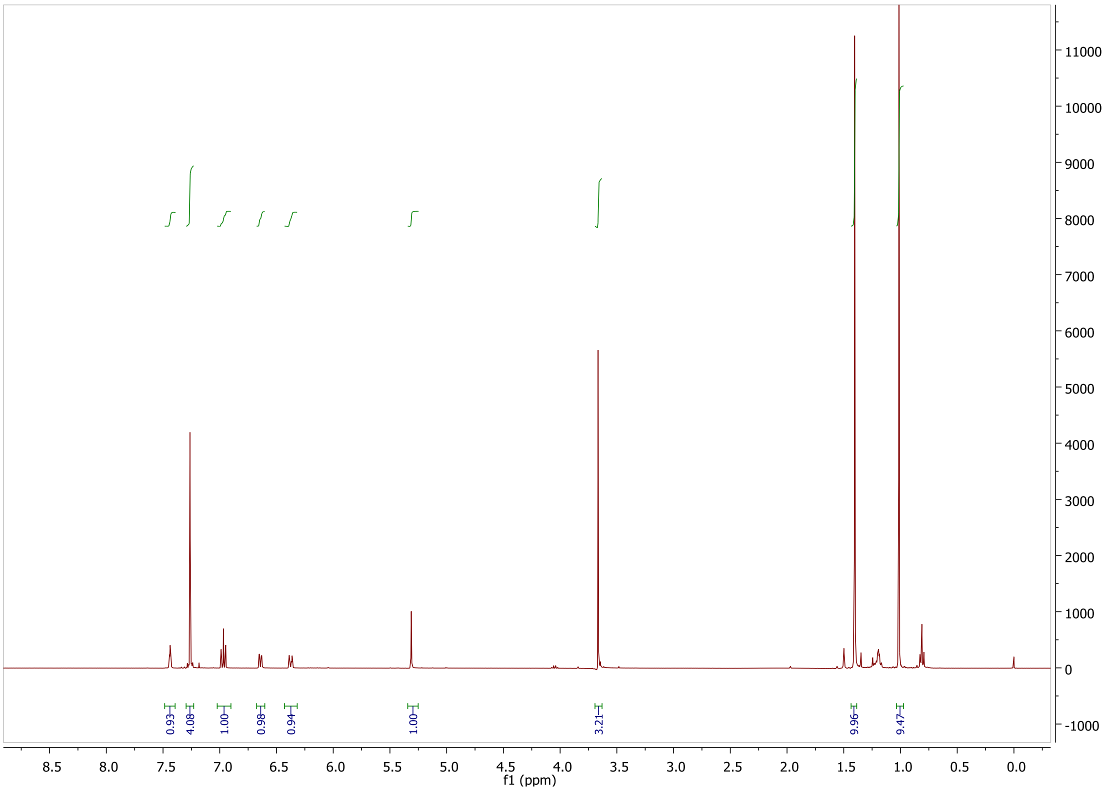

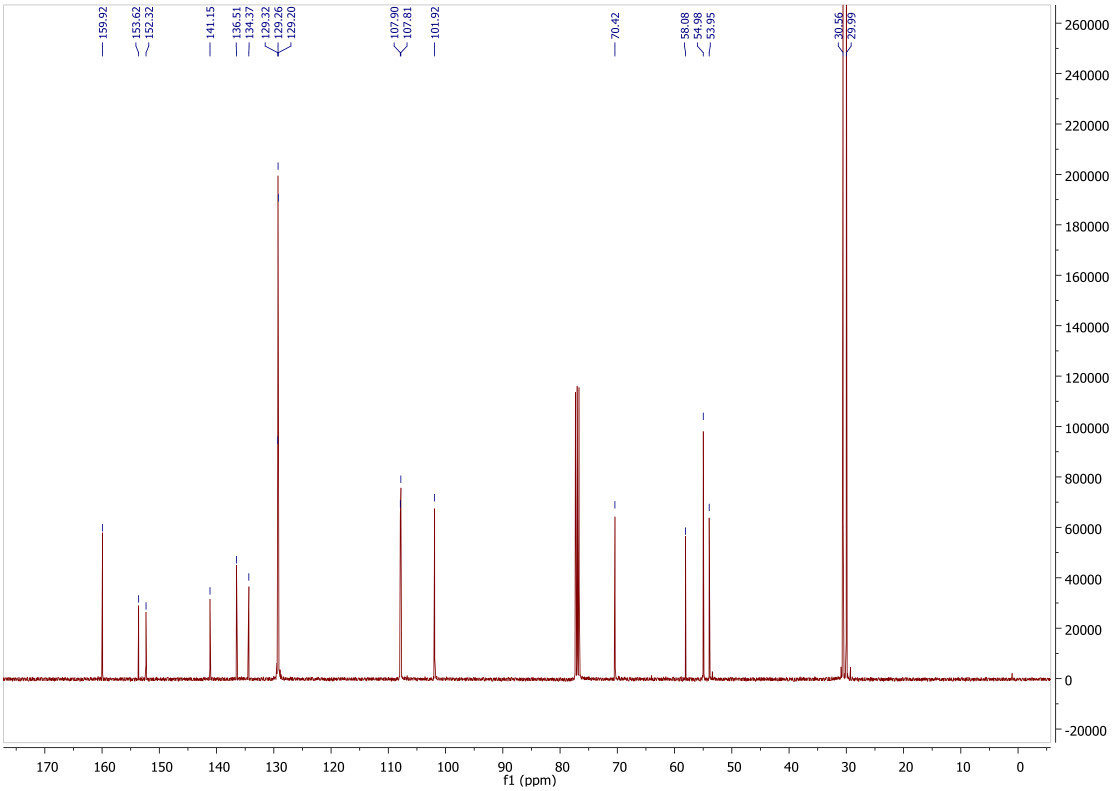

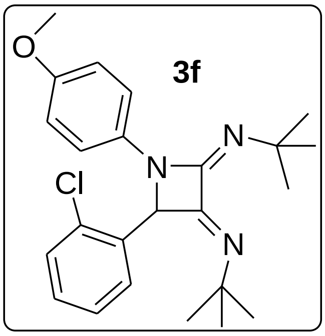

N,N'-(4-(2-Chlorophenyl)-1-(4-methoxyphenyl)azetidine-2,3-diylidene)bis(2-methylpropan-2-amine) **(3f)**

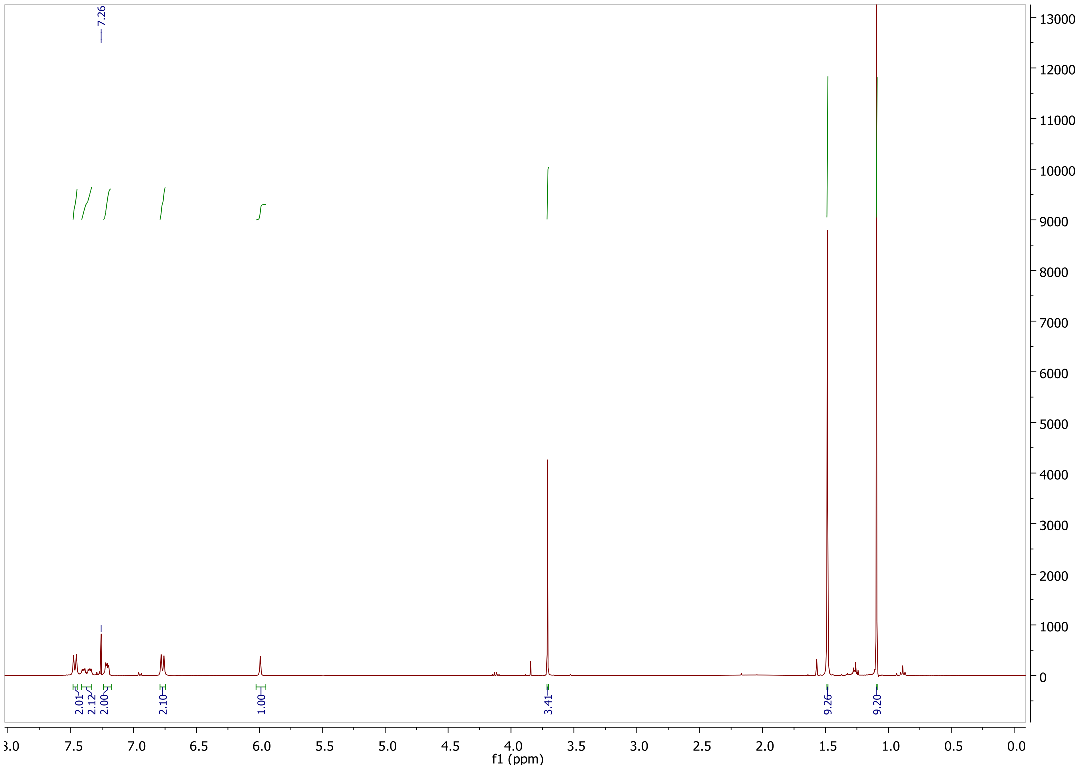

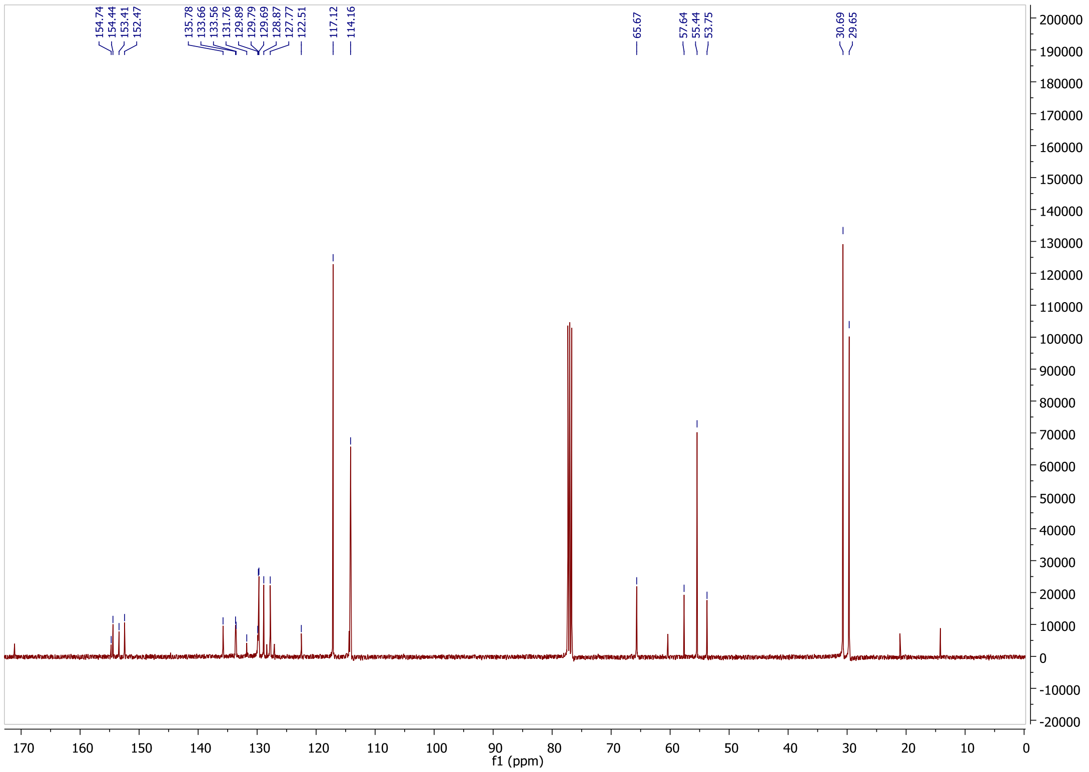

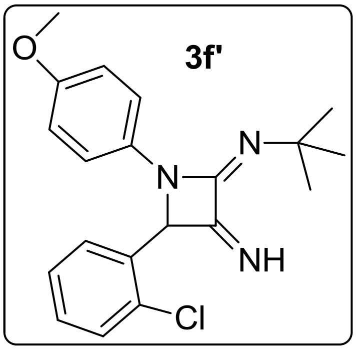

***N*-(4-(2-Chlorophenyl)-3-imino-1-(4-methoxyphenyl)azetidin-2-ylidene)-2-methylpropan-2-amine** (3f')

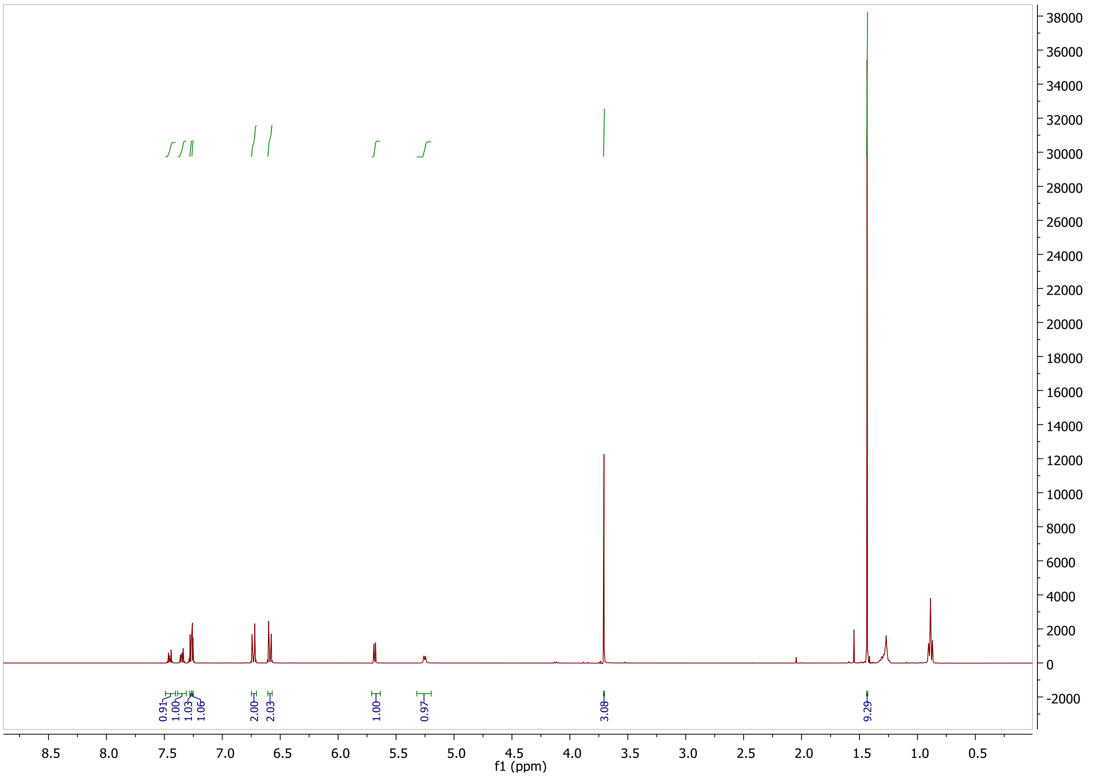

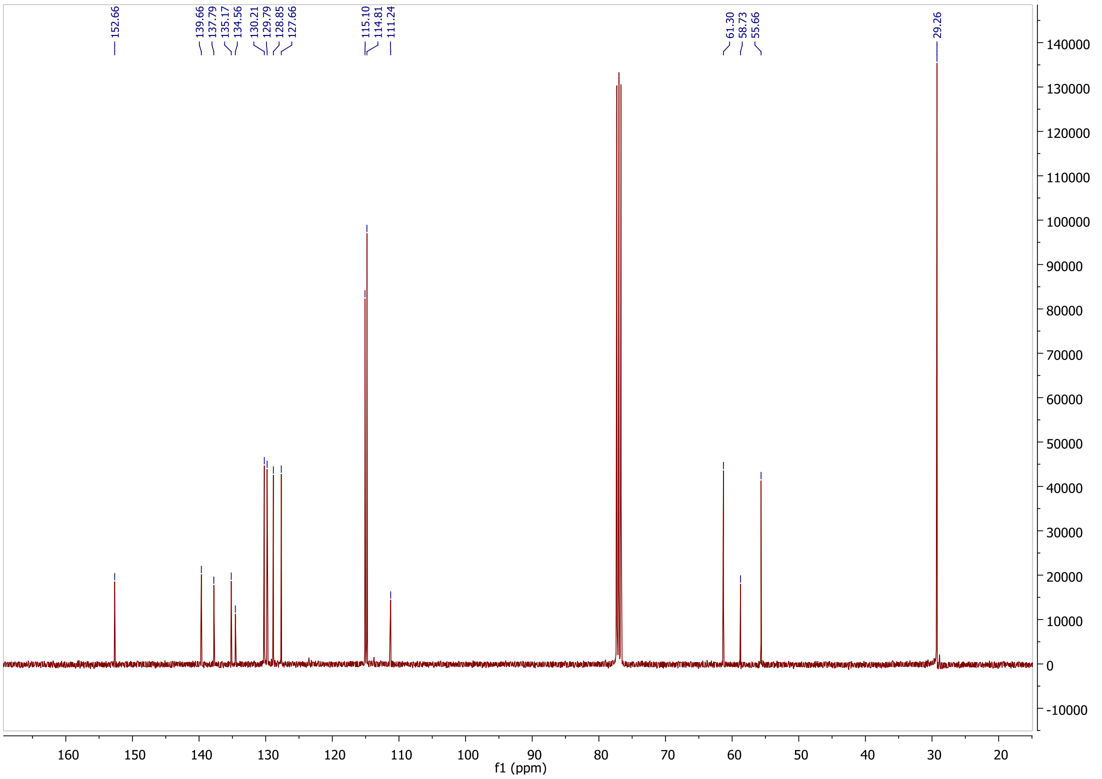

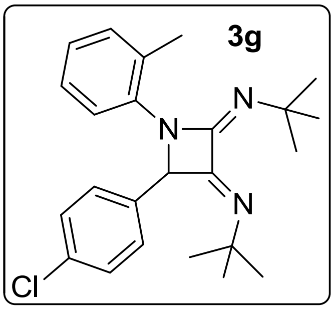

3g

***N,N'-(4-(4-Chlorophenyl)-1-o-tolylazetidine-2,3-diylidene)bis(2-methylpropan-2-amine)* (3g)**

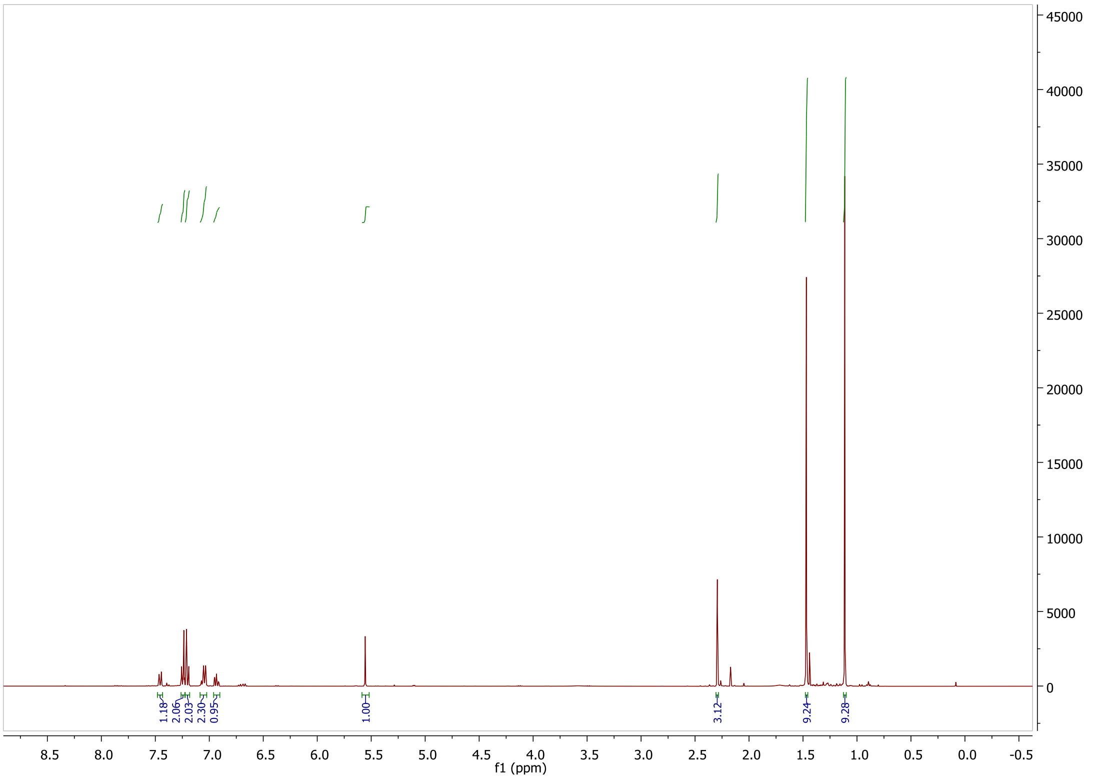

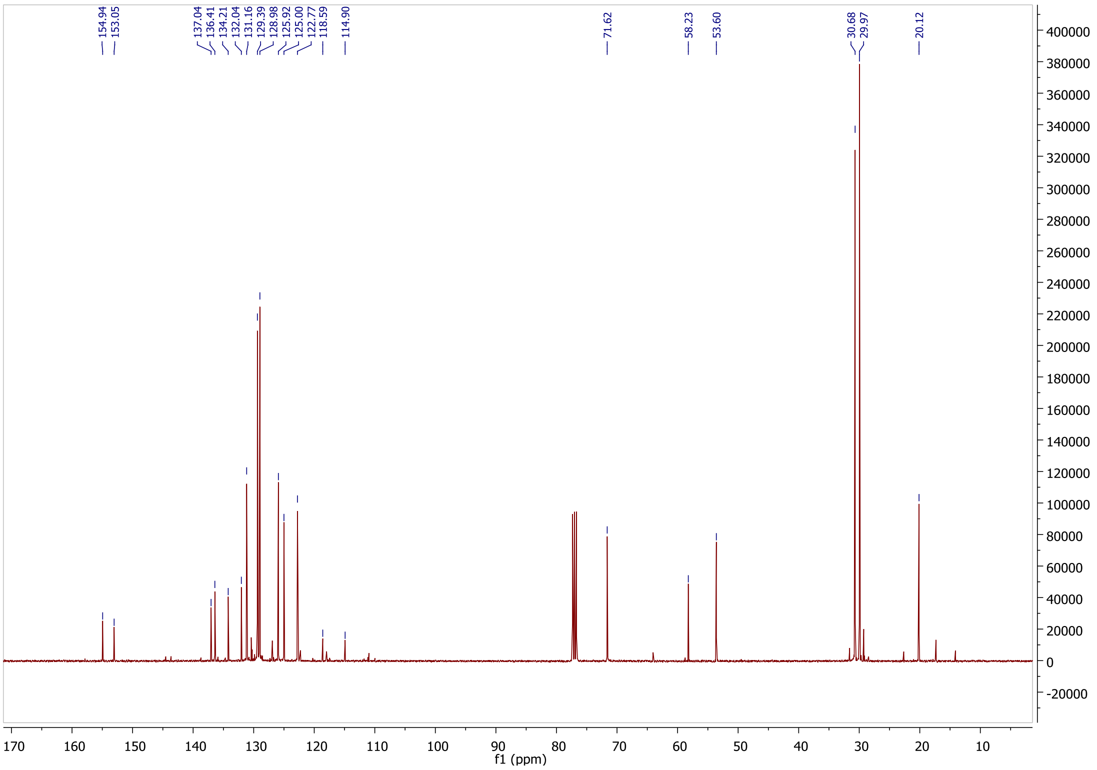

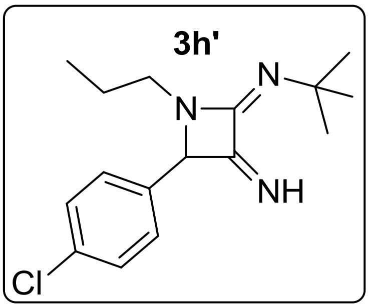

N-(4-(4-Chlorophenyl)-3-imino-1-propylazetidin-2-ylidene)-2-methylpropan-2-amine **(3h')**

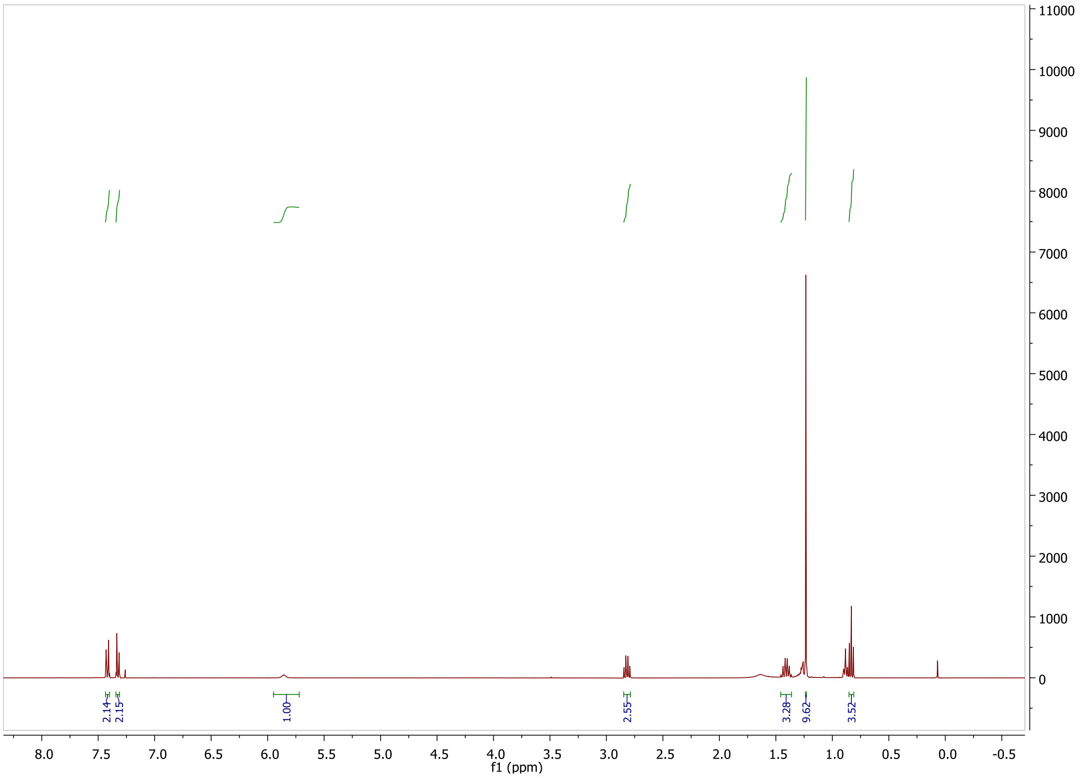

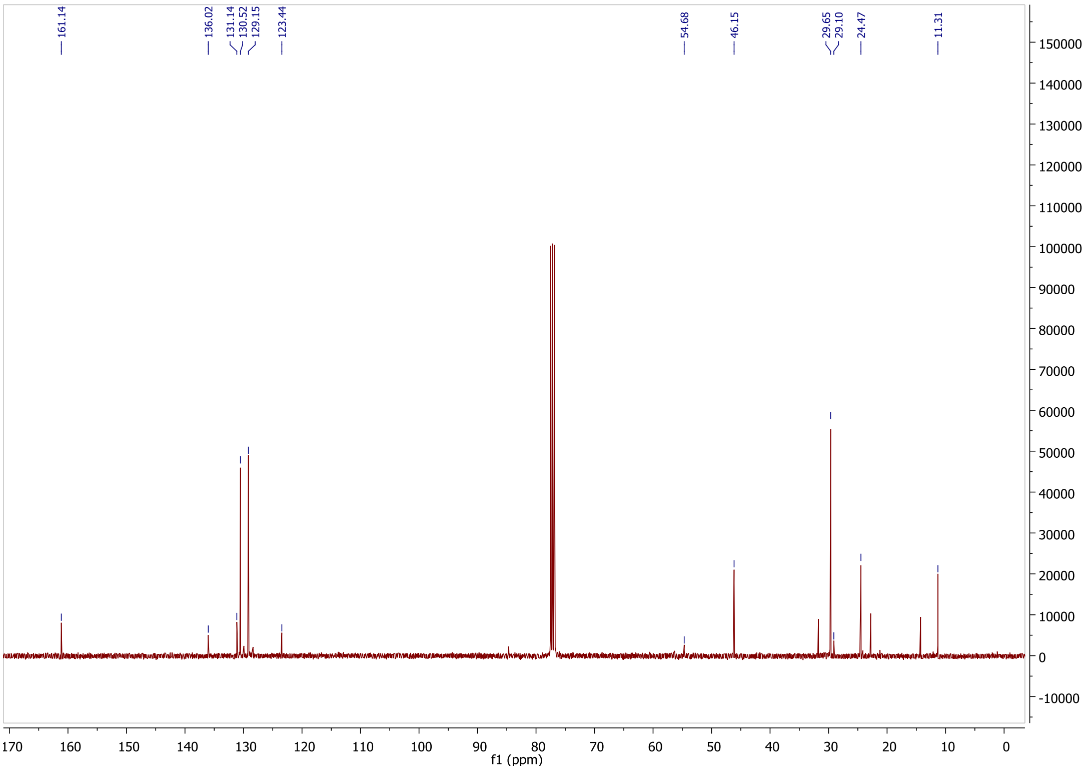

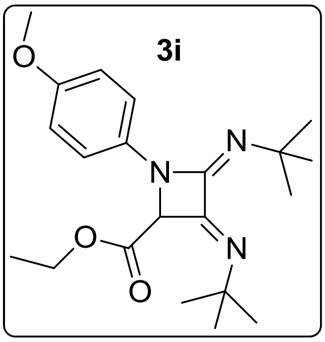

Ethyl 3,4-bis(tert-butylimino)-1-(4-methoxyphenyl)azetidine-2-carboxylate (3i)

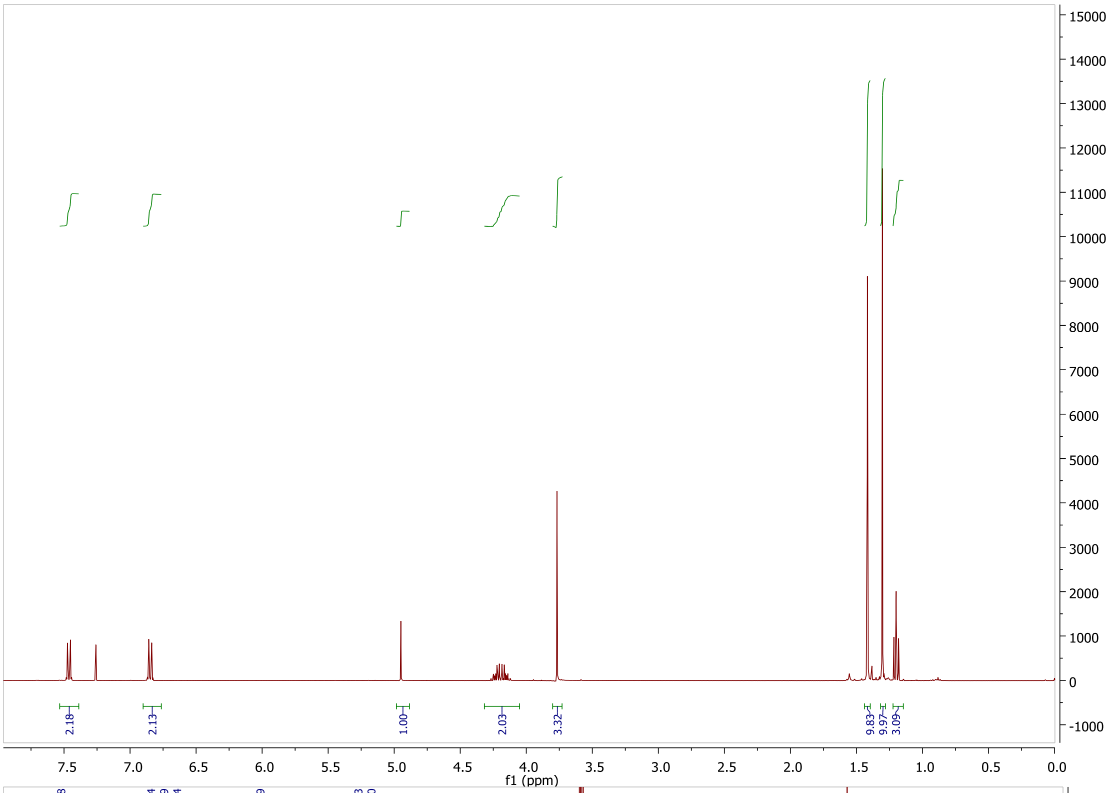

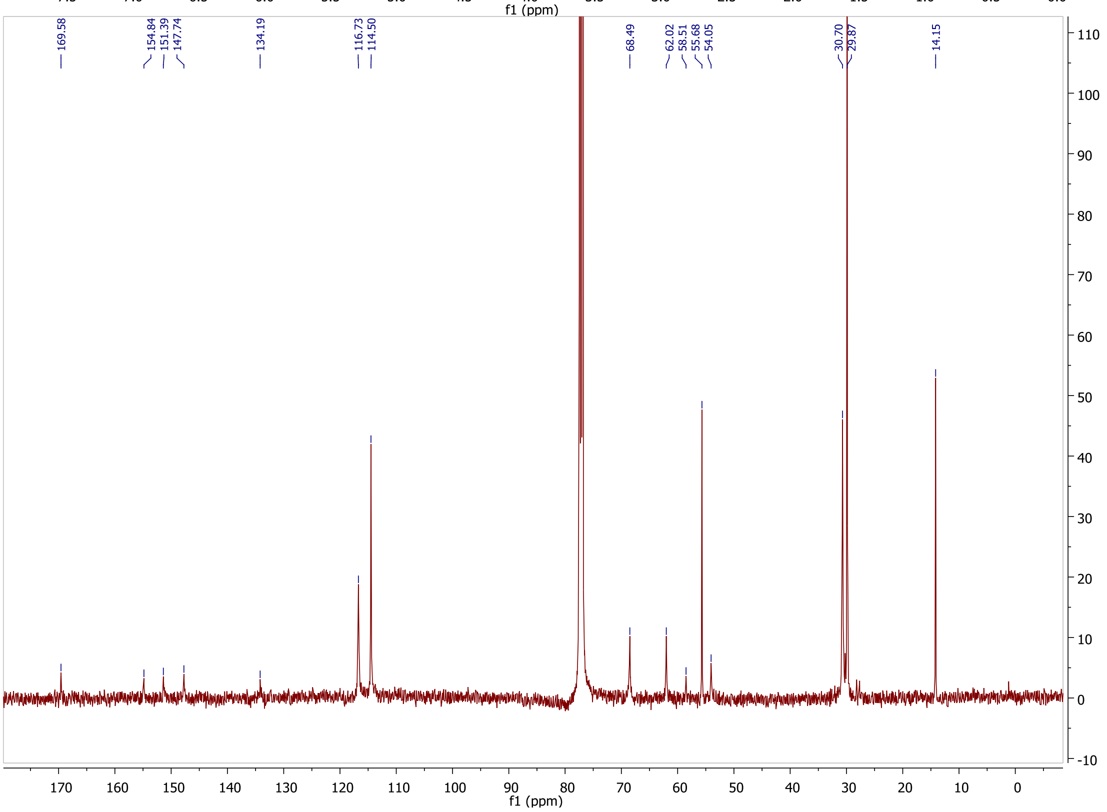

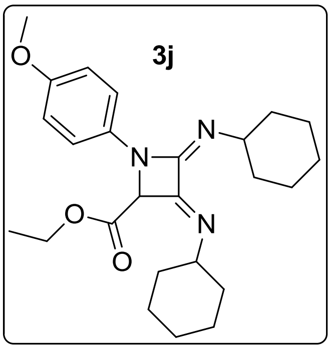

***Ethyl 3,4-bis(cyclohexylimino)-1-(4-methoxyphenyl)azetidine-2-carboxylate* (3j)**

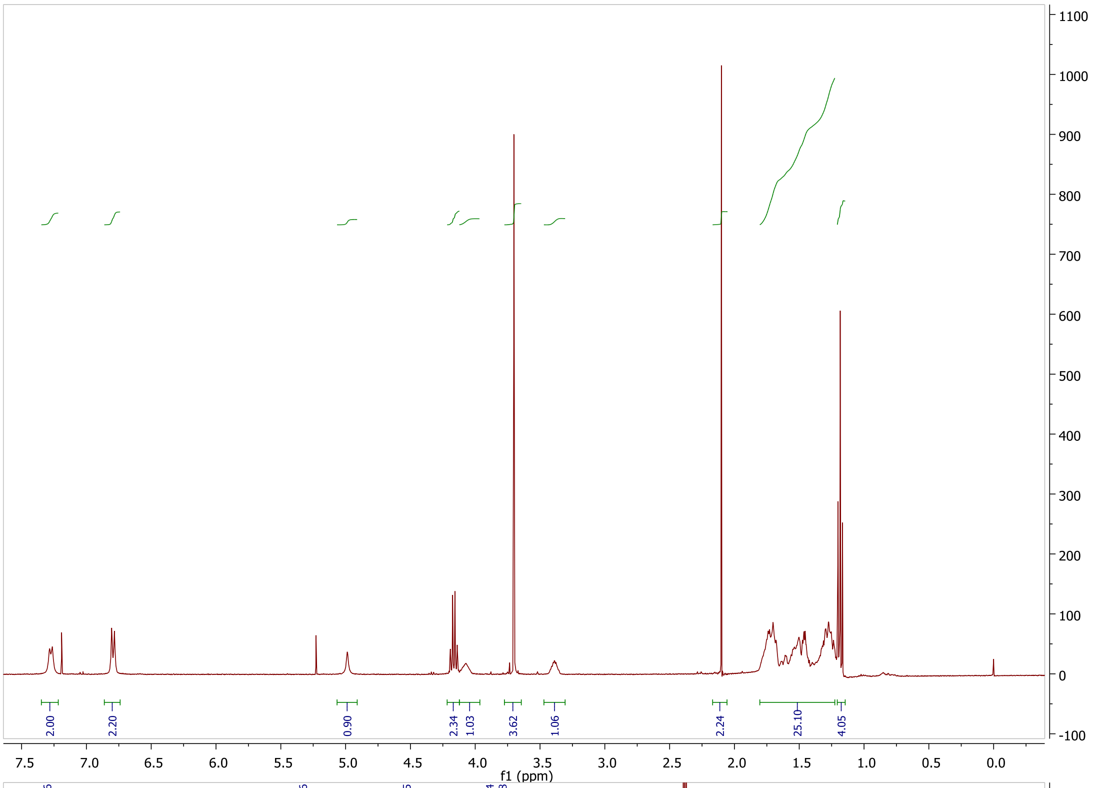

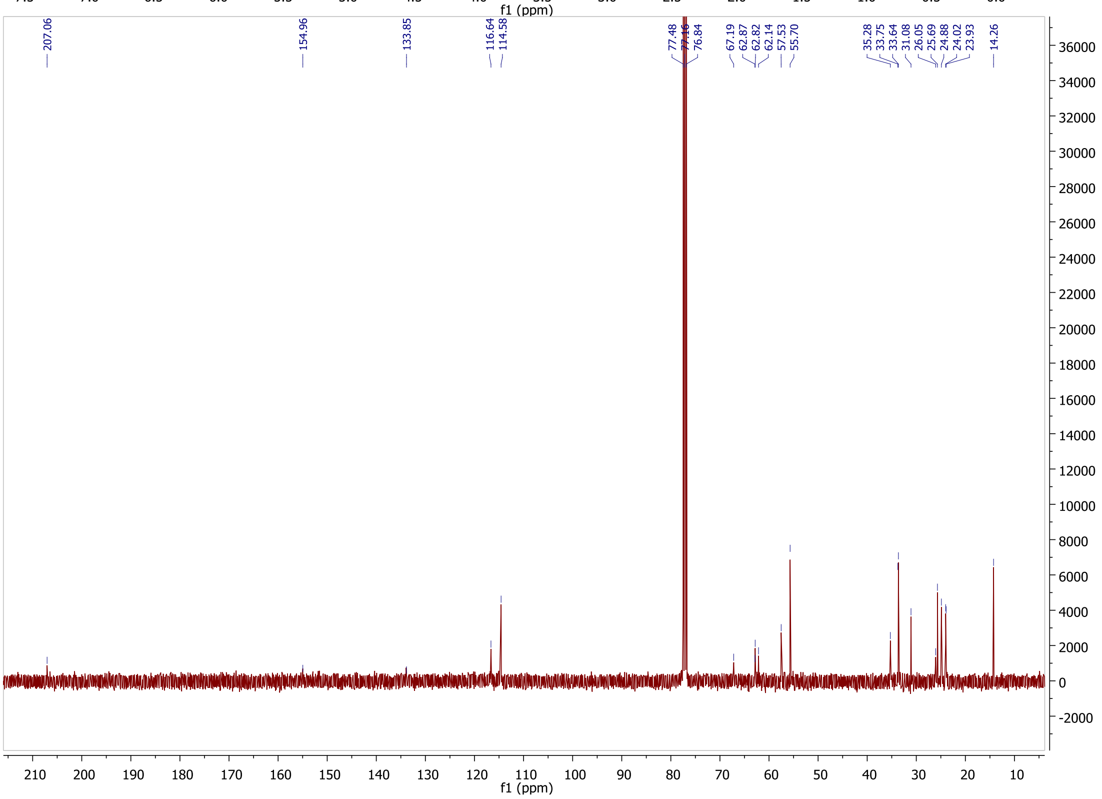

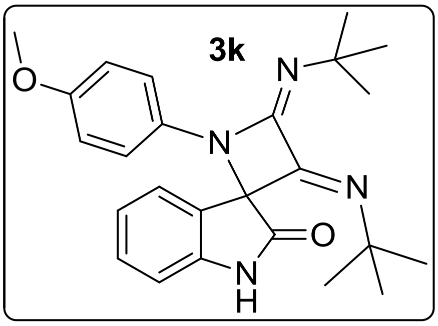

3,4-bis(tert-Butylimino)-1-(4-methoxyphenyl)spiro[azetidine-2,3'-indolin]-2'-one (3k)

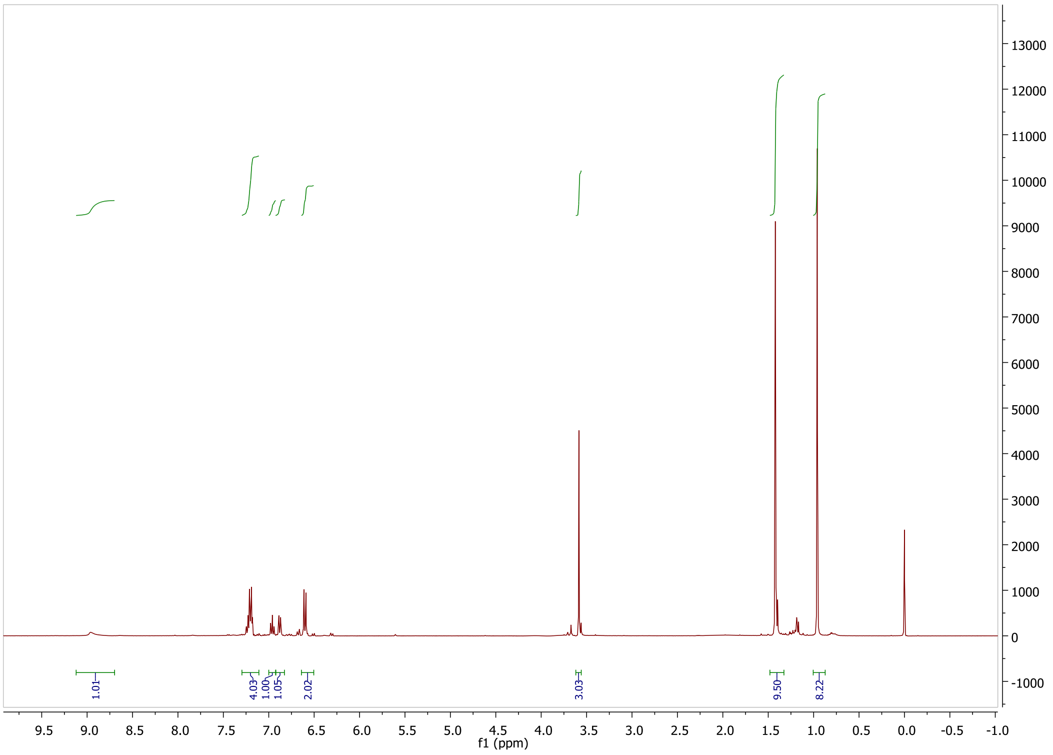

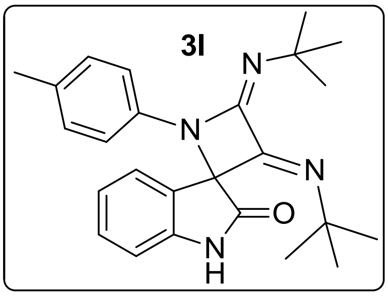

***3,4-bis(tert-Butylimino)-1-p-tolylspiro[azetidine-2,3'-indolin]-2'-one* (3l)**

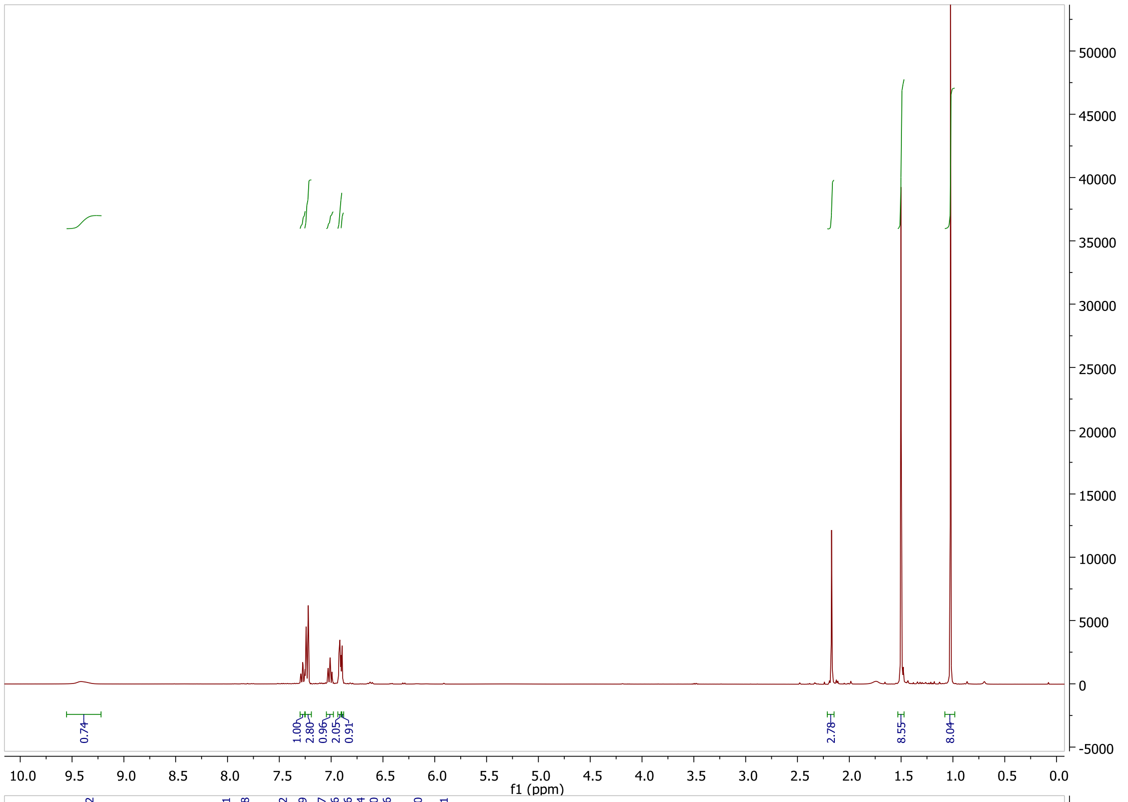

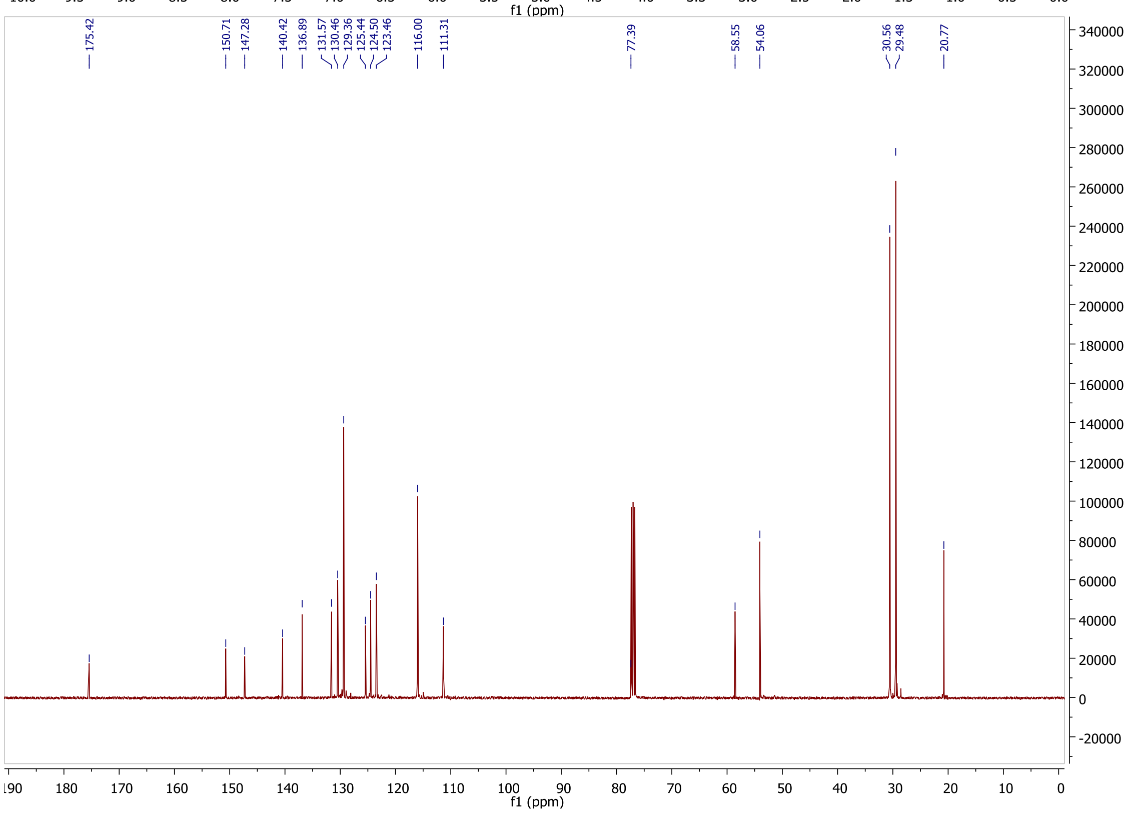

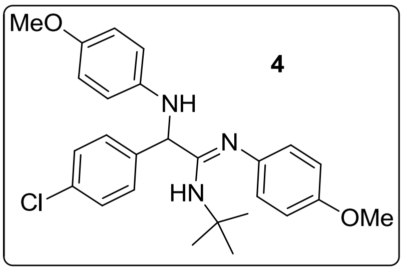

***N-(tert-Butyl)-2-(4-chlorophenyl)-N'-(4-methoxyphenyl)-2-((4-methoxyphenyl)amino)acetimidamide* (4)**

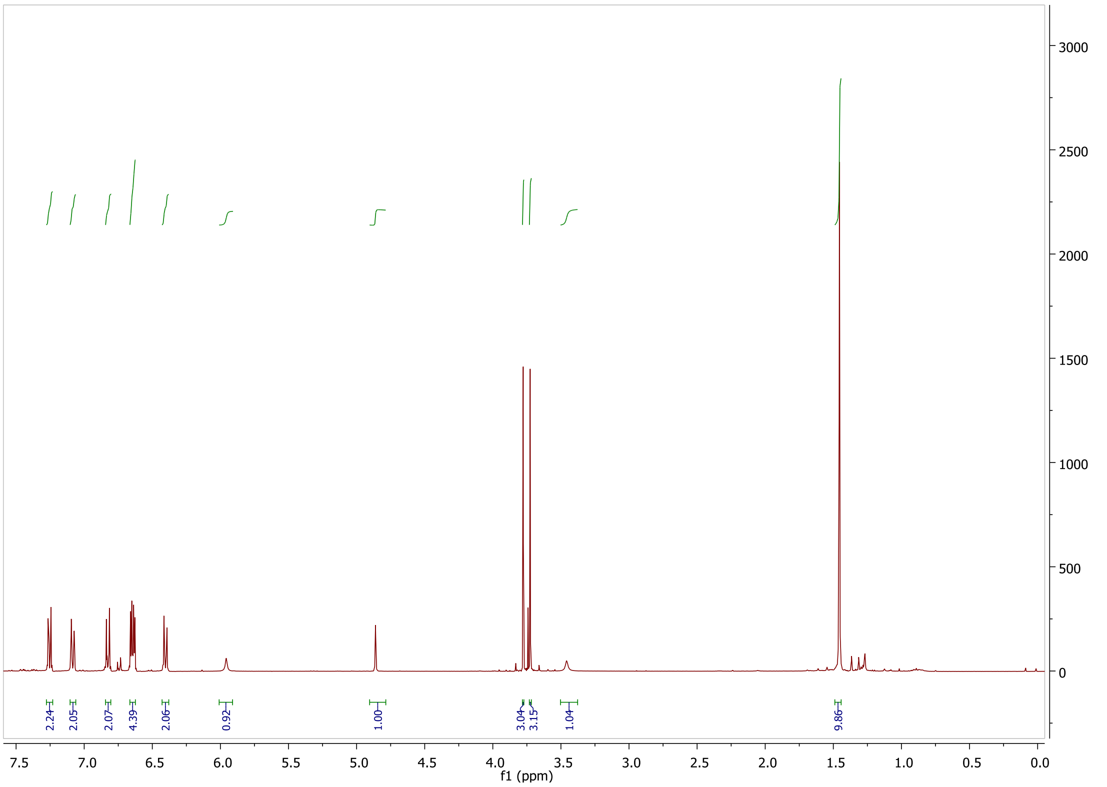

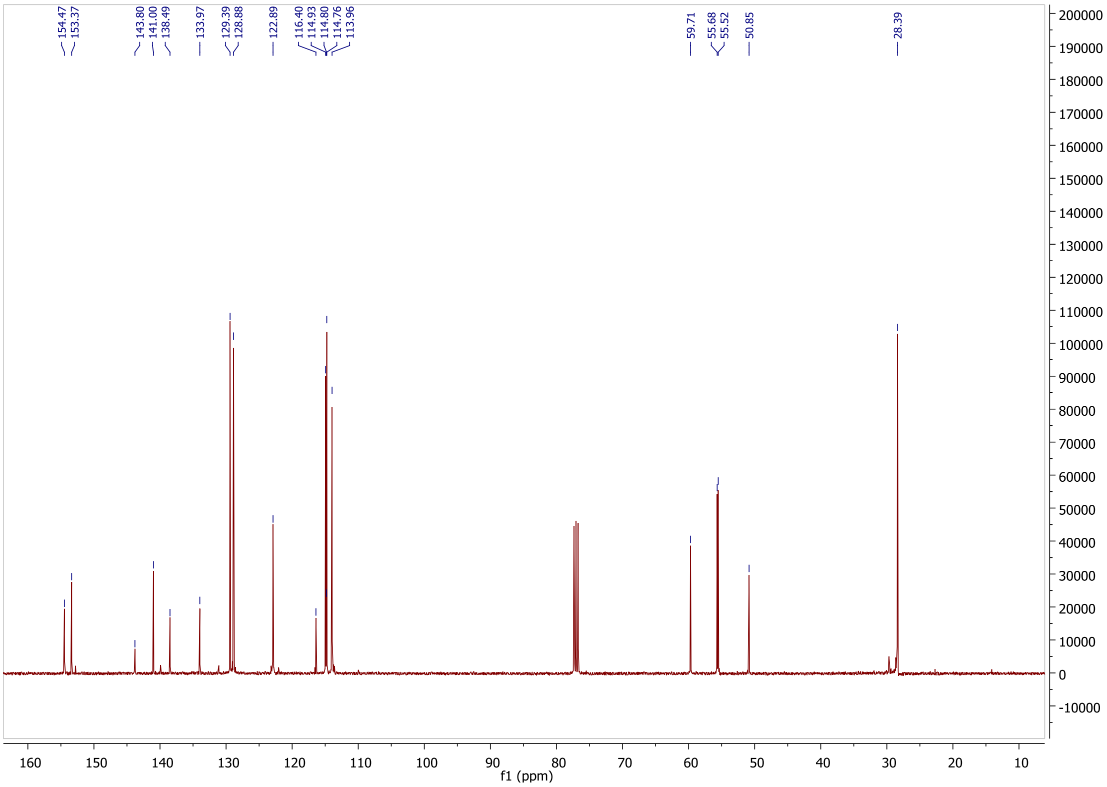

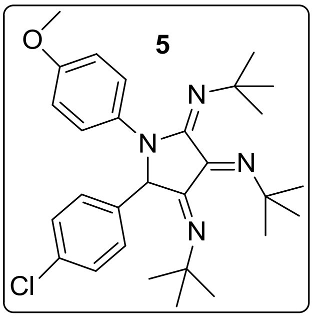

N,N',N''-(5-(4-Chlorophenyl)-1-(4-methoxyphenyl)pyrrolidine-2,3,4-triylidene)tris(2-methylpropan-2-amine) **(5)**

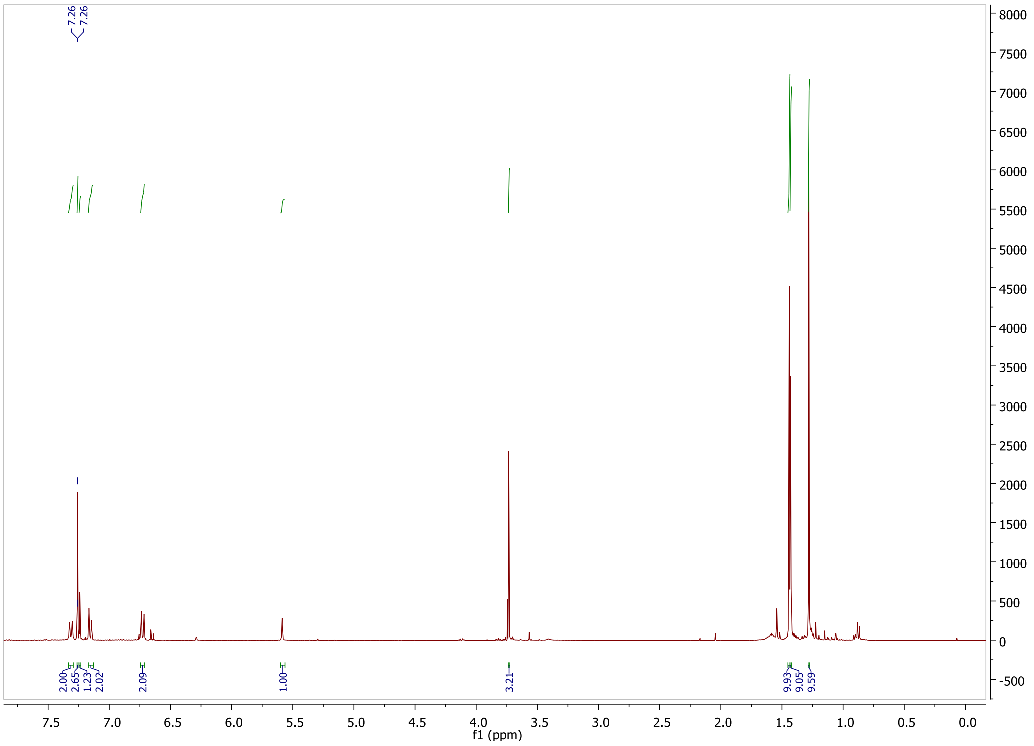

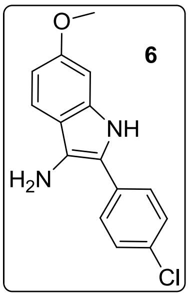

2-(4-Chlorophenyl)-6-methoxy-1H-indol-3-amine (6)

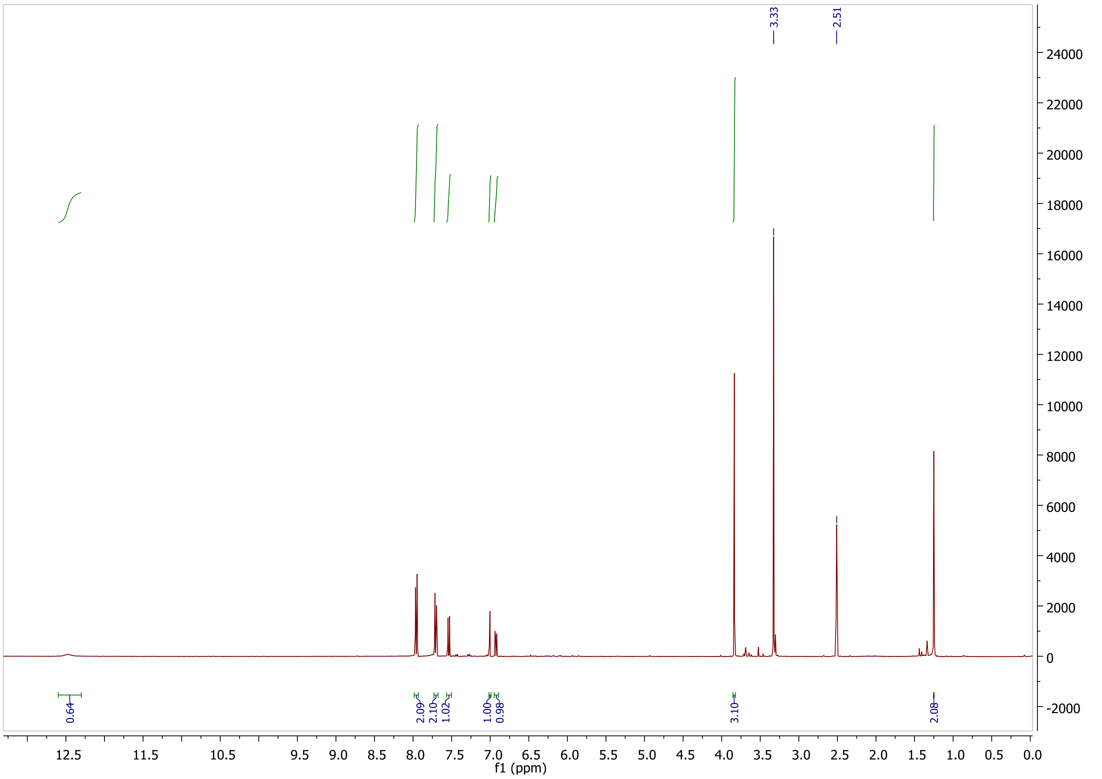

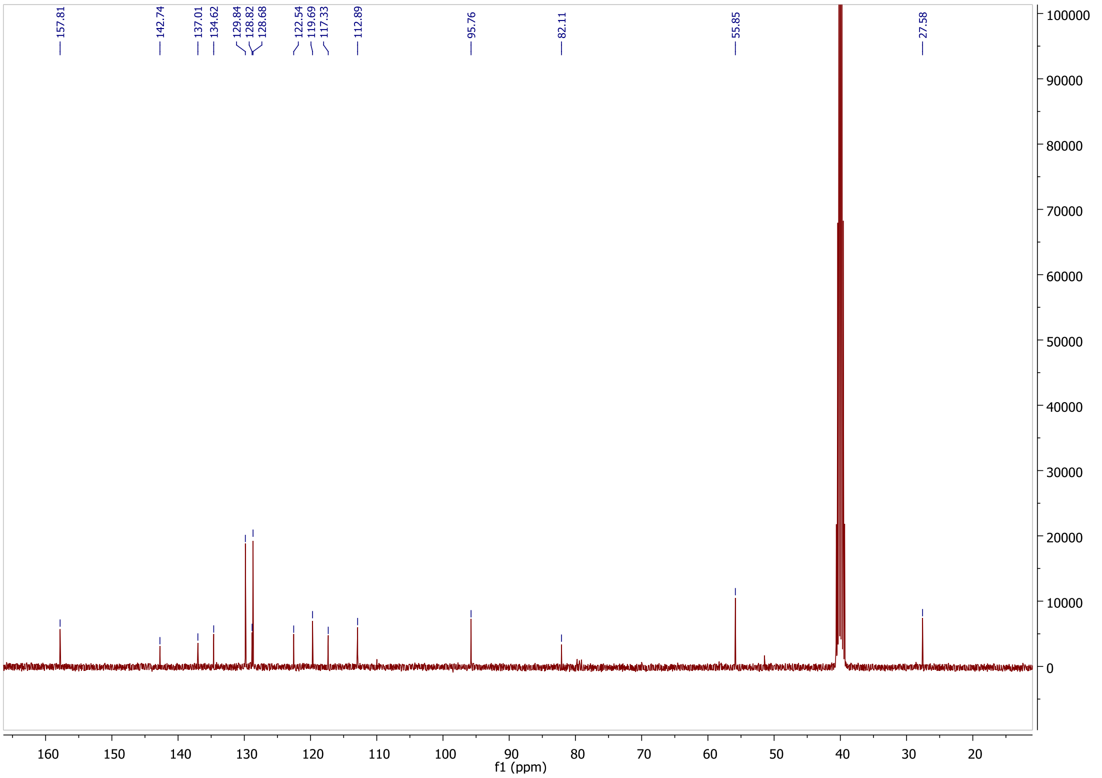

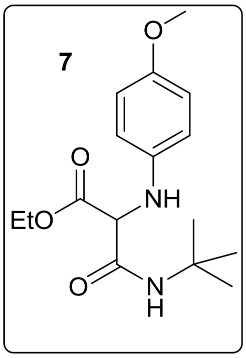

Ethyl 3-(tert-butylamino)-2-(4-methoxyphenylamino)-3-oxopropanoate **(7)**

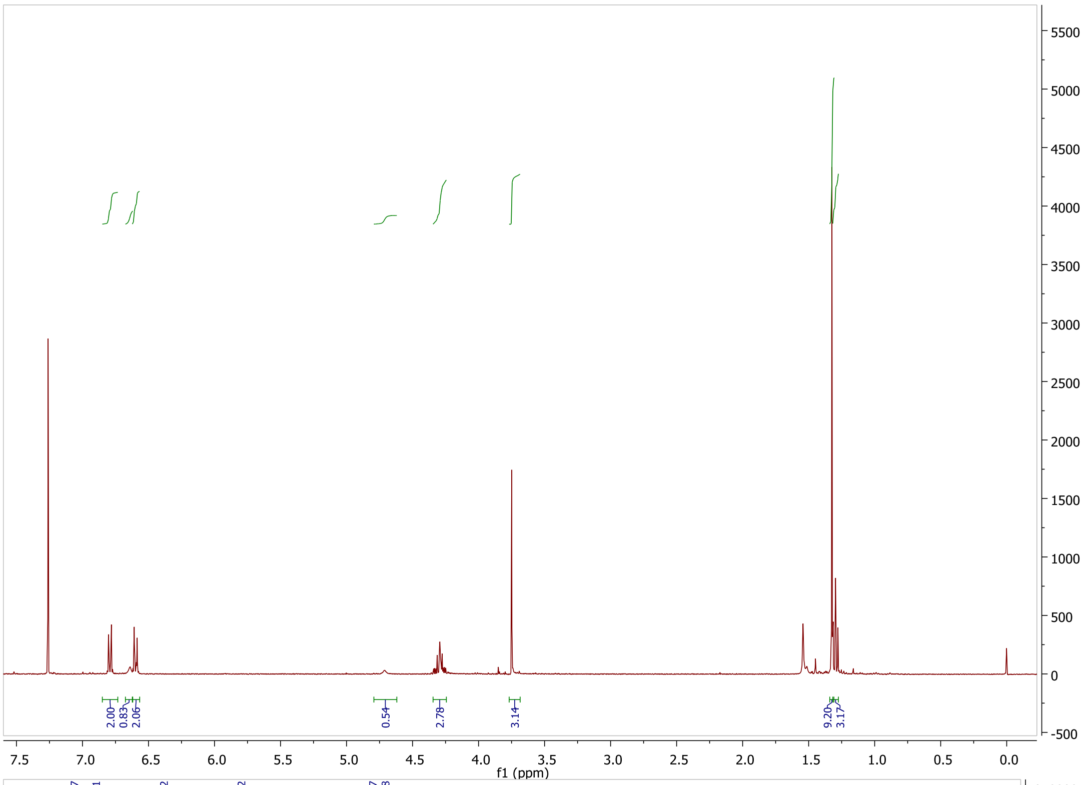

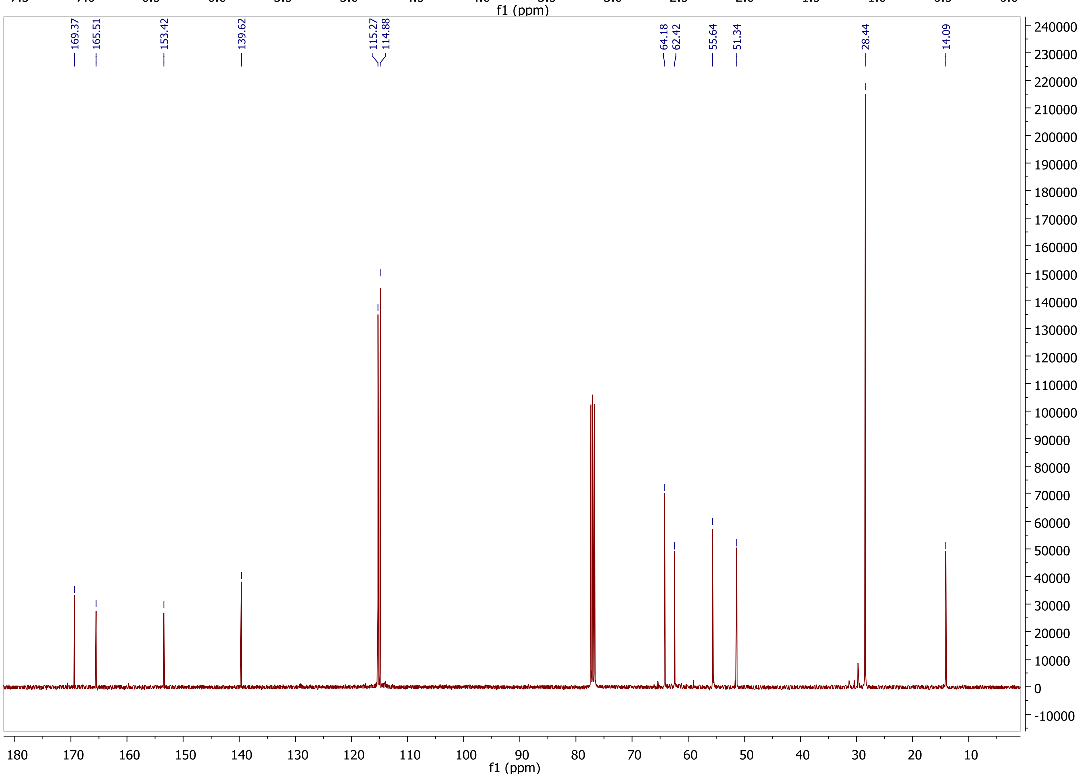

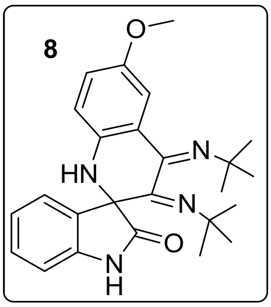

***3',4'-bis(tert-Butylimino)-6'-methoxy-3',4'-dihydro-1'H-spiro[indoline-3,2'-quinolin]-2-one* (8)**

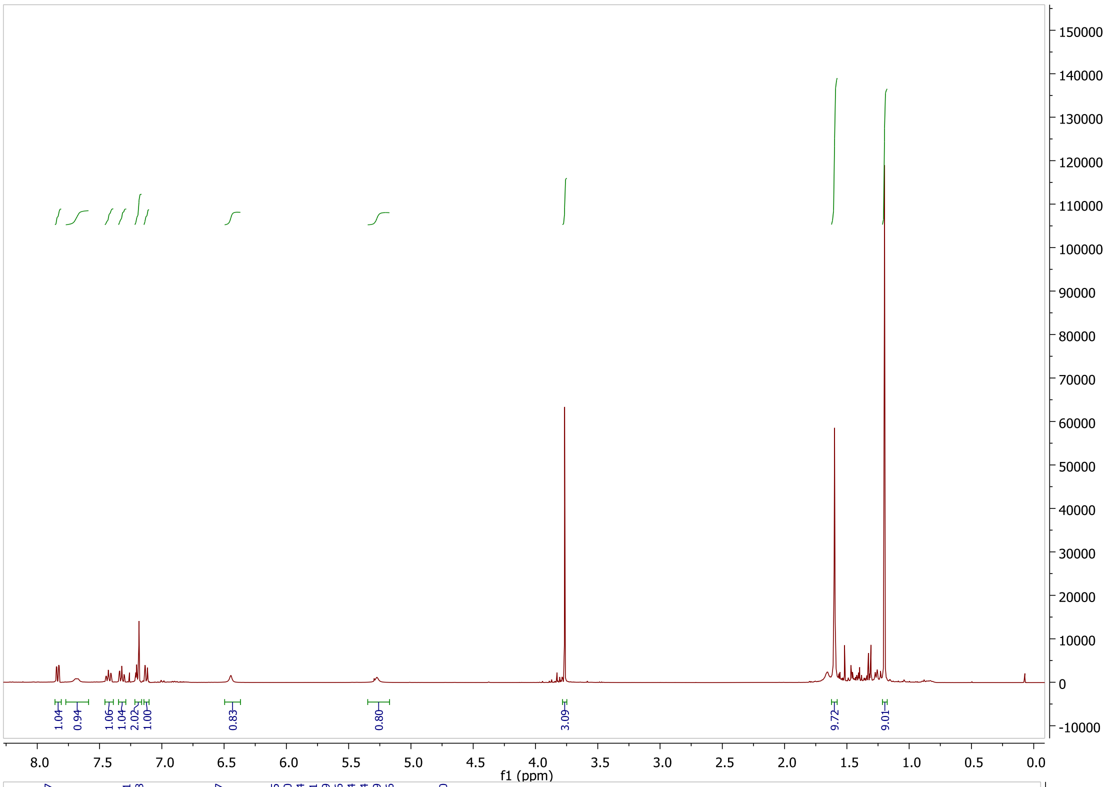

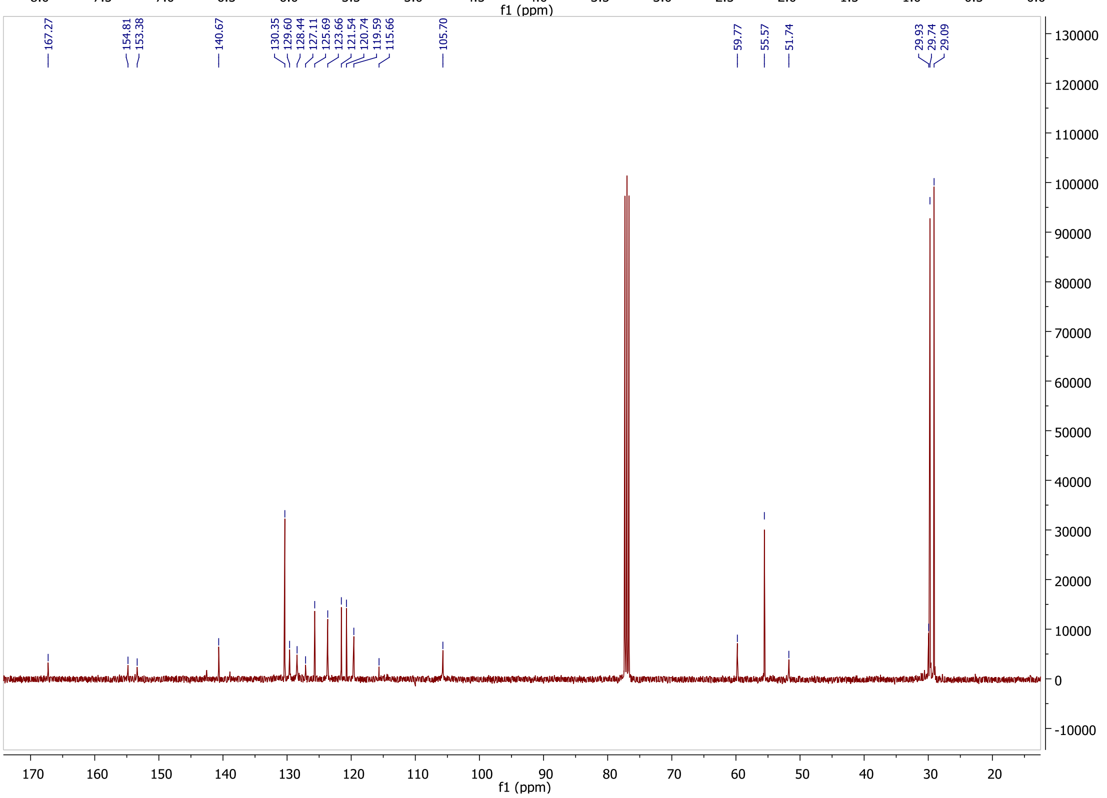

X-ray Structure of bis-aminoazetidine 3a

(CCDC 963354)

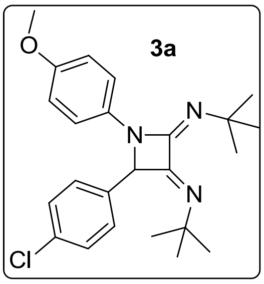

N,N'-(4-(4-Chlorophenyl)-1-(4-methoxyphenyl) azetidine -2,3-diylidene)bis(2-methylpropan-2-amine

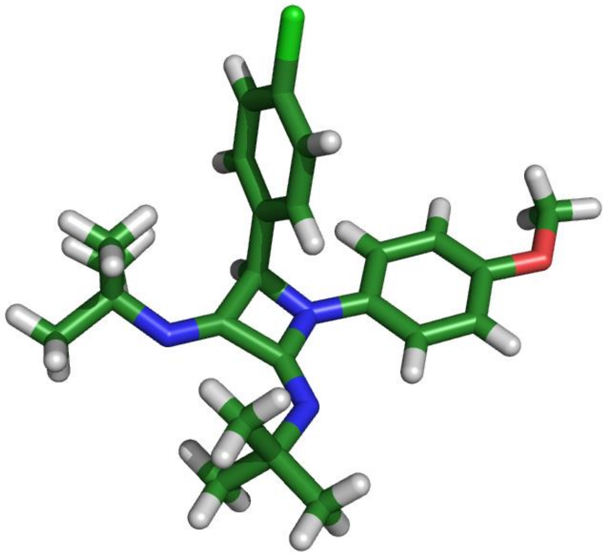

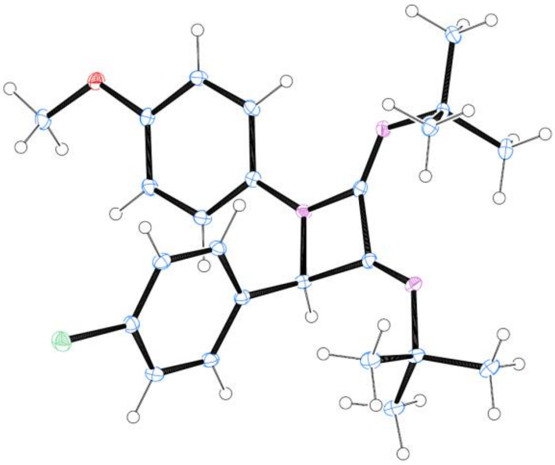

Cell views of bis-iminoazetidine **3a**

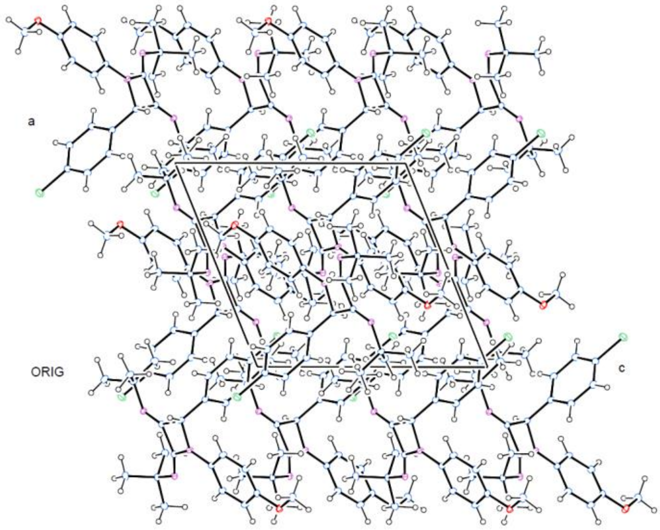

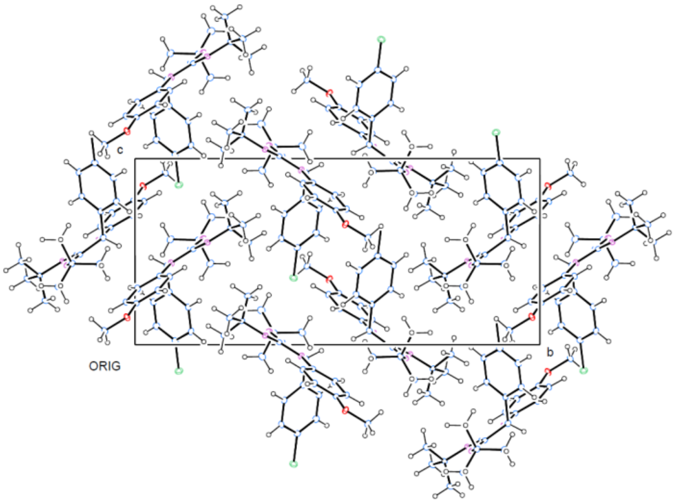

Supplement: File 1 — Experimental procedures, characterization data, copies of the NMR spectra for all new compounds and X-ray views of azetidine 3a. [file Beilstein_J_Org_Chem-10-12-s001.pdf]
